# The effects of cholesterol-lowering drugs on neurocognitive function: systematic review and meta analysis

**DOI:** 10.3389/fneur.2026.1696228

**Published:** 2026-02-04

**Authors:** Kaiwei Li, Ying Li, Xia Jiang, Ye Zhu

**Affiliations:** 1 Department of Cardiology, West China Hospital, Sichuan University, Chengdu, China; 2 Department of Cardiology, Chengdu Integrated TCM and Western Medicine Hospital, Chengdu, China; 3 Department of Cardiology, West China School of Public Health and West China Fourth Hospital, Sichuan University, Chengdu, China; 4 West China Hospital, Jincheng Hospital, Sichuan University, Chengdu, China

**Keywords:** cholesterol lowering drug, cholesterol-lowering drugs, dementia, low density lipoprotein cholesterol, neurocognitive function, statins, cholesterol absorption inhibitors, proprotein convertase subtilisin/kexin 9 inhibitors

## Abstract

**Objective:**

To investigate if cholesterol-lowering drugs exert effects on neurocognitive function.

**Methods:**

We searched Pubmed, Embase and Cochrane Libarary from inception to March 23rd, 2023, and searched clinicaltrials.gov on January 23rd, 2024. Randomized controlled trials that evaluated neurocognitive events and neurocognitive function after using cholesterol-lowering drugs including statins, cholesterol absorption inhibitors, proprotein convertase subtilisin/kexin 9 inhibitors were collected. The literature screening, data extraction and quality evaluation were carried out independently by two researchers, and the random effect model was used to pool the data.

**Results:**

A total of 42 studies with 150,405 subjects were included. Cholesterol-lowering drugs did not increase the risk of neurocognitive events (RR: 0.99, 95% CI: 0.88–1.12). Subgroup analysis by the type of drugs did not suggest that statins (RR: 0.94, 95% CI: 0.72–1.25), ezetimibe (RR: 1.11, 95% CI: 0.71–1.74) or proprotein convertase subtilisin/kexin 9 inhibitors (RR: 1.00, 95% CI: 0.87–1.14) increased the risk of neurocognitive events. By pooling the outcomes of the neurocognitive test scale, we found that cholesterol-lowering drugs did not change neurocognitive function in the five domains of attention, psychomotor speed, executive function, working memory and memory, as well as global effect.

**Conclusion:**

Cholesterol-lowering drugs including statins, cholesterol absorption inhibitors and PCSK9 inhibitors have no adverse effects on neurocognitive function. The decrease of low-density lipoprotein cholesterol will not lead to the decline of neurocognitive function.

**Systematic review registration:**

https://www.crd.york.ac.uk/PROSPERO/view/CRD42023404802, Identifier: CRD42023404802

## Introduction

Atherosclerotic cardiovascular disease (ASCVD) has emerged as a major global health threat. Over the past few decades, the burden of ASCVD in China has increased rapidly (1). Elevated levels of plasma low-density lipoprotein cholesterol (LDL-C) are recognized as the primary risk factor for ASCVD (2), and LDL-C has become the primary therapeutic target established in major clinical guidelines for the prevention and management of ASCVD. Currently, three major classes of LDL-C-lowering medications are available in the market, statins, cholesterol absorption inhibitors, and proprotein convertase subtilisin/kexin type 9 (PCSK9) inhibitors. Statins exert their pharmacological effect by inhibiting cholesterol synthesis through the inhibition of 3-hydroxy-3-methylglutaryl-coenzyme A (HMG-CoA) reductase (3). Cholesterol absorption inhibitors exert their mechanism of action by inhibiting the sterol transporter Niemann-Pick C1-Like 1 (NPC1L1), thereby reducing cholesterol absorption in the small intestine (4). PCSK9 inhibitors inhibit the interaction between PCSK9 and LDL-C receptors on hepatocyte membranes, thereby enhancing the hepatic clearance of LDL-C (5). The Scandinavian Simvastatin Survival Study (4S) (6) demonstrated that patients receiving simvastatin achieved a 35% reduction in LDL-C from baseline, whereas those in the placebo group experienced a 1% increase. The IMPROVE-IT trial (7) showed that the addition of ezetimibe to statin therapy resulted in a further 24% reduction in LDL-C at 1 year. Similarly, the ODYSSEY OUTCOMES study (8) confirmed that alirocumab, administered in addition to statin therapy, resulted in a 45.7% reduction in LDL-C from baseline, compared with an 8% increase in the placebo group.

Although the cardiovascular benefits of cholesterol-lowering therapies are well established, controversy persists regarding whether reduced cholesterol levels in neurons, neurotransmitters, and myelin sheaths may adversely affect brain function. Cholesterol is a vital biological molecule and a primary structural component of cell membranes. Notably, the brain is highly enriched in cholesterol, accounting for approximately 25% of the total cholesterol in the human body (9). Cholesterol plays a critical role in maintaining the structural integrity of cell membranes, which leads to continued scientific debate regarding the potential effects of LDL-C reduction on neuronal structure and function (10). As a result, the potential impact of cholesterol-lowering interventions on neurocognitive function has garnered significant attention in recent research.

More than twenty years ago, case reports first suggested a possible association between the onset of memory impairment in patients and the administration of statin medications (11). Patients experienced memory decline during statin therapy, which resolved after discontinuation but recurred following rechallenge. An observational study (12) examined 171 self-reported cases of neurocognitive impairments potentially associated with statin use, employing the Naranjo adverse drug reaction probability scale. The study found that 75% of patients experienced neurocognitive impairments associated with statin use. Among 143 patients who discontinued statin therapy, 128 demonstrated improvement in cognitive impairments; however, among the 29 patients who resumed statin treatment, 19 experienced recurrence of neurocognitive impairments. Randomized controlled trials have also demonstrated that statin therapy may be associated with declines in neurocognitive function. Muldoon et al. (13, 14) demonstrated adverse effects of lovastatin and simvastatin on specific domains of neurocognitive function. Conversely, other studies suggested that statins have no adverse impact on neurocognitive function. Two large randomized controlled trials, PROSPER and HPS, found that neither pravastatin nor simvastatin induced cognitive decline in study participants (15, 16). With the emergence of novel cholesterol-lowering therapies such as PCSK9 inhibitors, it is now possible to achieve further reductions in LDL-C levels (17). A safety study of PCSK9 monoclonal antibodies indicated a potential increased risk of neurocognitive impairment (18).

Given the lack of consensus on whether cholesterol-lowering agents may be associated with declines in neurocognitive function, this study aims to conduct a systematic review and meta-analysis to assess the effects of various lipid-lowering drug classes on neurocognitive performance, with the objective of generating evidence-based conclusions.

## Methods

### Study design

This systematic review and meta-analysis follows the PRISMA statement guidelines for reporting (19). The protocol was registered in PROSPERO (CRD42023404802).

### Data sources

We conducted searches on PubMed, Embase, and Cochrane Library, from inception to March 23rd, 2023, with language restricted to English. To ensure the inclusion of the most recent literature, we also searched clinical trial registries (clinicaltrials.gov) on January 23rd, 2024. The search terms used primarily included: ((Hydroxymethylglutaryl-CoA Reductase Inhibitors) OR (Statins) OR (Ezetimibe) OR (PCSK9 Inhibitors) OR (Alirocumab) OR (Evolocumab) OR (Inclisiran)) AND (randomized controlled trial). The complete search strategy is provided in Appendix 1.

### Study selection

We selected the trials using the following inclusion and exclusion criteria. We included trials if (1) it was a randomized controlled trial with a minimum follow-up period of 24 weeks; (2) Intervention involved statins, cholesterol absorption inhibitors, or PCSK9 inhibitors; (3) Control measures included placebo, no treatment, standard therapy, or a different type of cholesterol-lowering medication compared to the intervention; (4) it reported neurocognitive outcomes before and after the intervention. We included trials if (1) it was an animal trial, an *in vitro* trial, or a phase I clinical trial; (2) populations were affected by neurocognitive diseases (such as Alzheimer’s disease, schizophrenia); (3) it involved the PCSK9 inhibitor bococizumab, which had been withdrawn from the market due to neutralizing antibodies leading to diminished efficacy.

Two researchers performed the study selection independently. Discrepancies were resolved through discussion with a third researcher. Initial screening was based on titles and abstracts, followed by full-text review of potentially relevant articles to determine final inclusion. For studies involving duplicate populations, priority was given to those with the longest follow-up duration, followed by studies with the largest sample sizes.

### Data extraction

Two researchers independently extracted data, resolving discrepancies through discussion with a third researcher. Using pre-designed forms, data extracted from included literature included: study title/first author, publication year, characteristics of study population, age, intervention measures, control measures, sample size, follow-up duration, cognitive outcomes, and pre-post intervention mean and standard deviation of LDL-C levels.

For studies lacking reported data, information was supplemented from clinicaltrials.gov and related literature. In cases of multiple intervention or control groups, data from the group with the largest sample size were prioritized for selection. Subsequently, the intervention group with the greatest reduction in LDL-C levels was chosen, while the group with the smallest reduction served as the control. Regarding post-treatment LDL-C data, priority was given to the most complete data reported during follow-up, followed by average LDL-C data during follow-up or data from the longest follow-up period.

Referring to several relevant clinical studies and meta-analyses (20–22), we defined neurocognitive events as confusion, amnesia, memory impairment, attention disorders, dementia, disorientation, frontotemporal dementia, transient global amnesia, cognitive impairment, delirium, Alzheimer’s disease, aphasia, reading disorders, vascular encephalopathy, hallucinations, and mental status change. We categorized neurocognitive test scales into six domains for assessing different aspects of cognition: attention, psychomotor speed, executive function, working memory, memory, and global effect (14, 23, 24).

### Quality assessment

Two researchers independently assessed the quality of studies, consulting a third researcher in cases of disagreement. Cochrane bias risk assessment tools were used to evaluate literature bias risk (25), covering random sequence generation, allocation concealment, blinding of participants and personnel, blinding of outcome assessment, incomplete of outcome data, selective reporting, and other bias. Each aspect was rated as low risk, unclear, or high risk.

### Statistical analysis

For the incidence rate of neurocognitive events, we conducted an analysis using binary data and applied the random-effect model to calculate the relative risk (RR) and its 95% confidence interval (CI), while for neurocognitive test scores, we conducted an analysis using continuous data and applied the random-effect model to calculate standardized mean differences (SMD) and its 95% CI. For tests where lower scores indicate better cognitive function, results were inverted (multiplied by −1) so that higher scores reflected better cognitive function. Differences between treatment groups in different cognitive domains were separately analyzed. In cases where mean values were unreported, estimates were calculated using median and interquartile range, while standard errors and CIs were used in the absence of reported standard deviations (26).

Heterogeneity was assessed using *Q*-tests and *I*^2^ statistics, with *Q*-test *p* > 0.1 and *I*^2^ ≤ 50% indicating low heterogeneity. Sensitivity analyses were performed by changing effect models and sequentially excluding literature. Subgroup analyses included cholesterol-lowering drug types, study populations, follow-up times, and studies specifically including participants aged ≥60 years. Meta-regression was employed to explore dose–response relationships between the occurrence risk of neurocognitive events and absolute and percentage changes in LDL-C before and after intervention. Publication bias was assessed using funnel plots, Begg’s test, and Egger’s test, with *p* ≥ 0.1 indicating no publication bias. All statistical analyses were conducted using Stata 16.0 and Revman 5.3 software.

## Results

### Included studies

**Figure 1:**
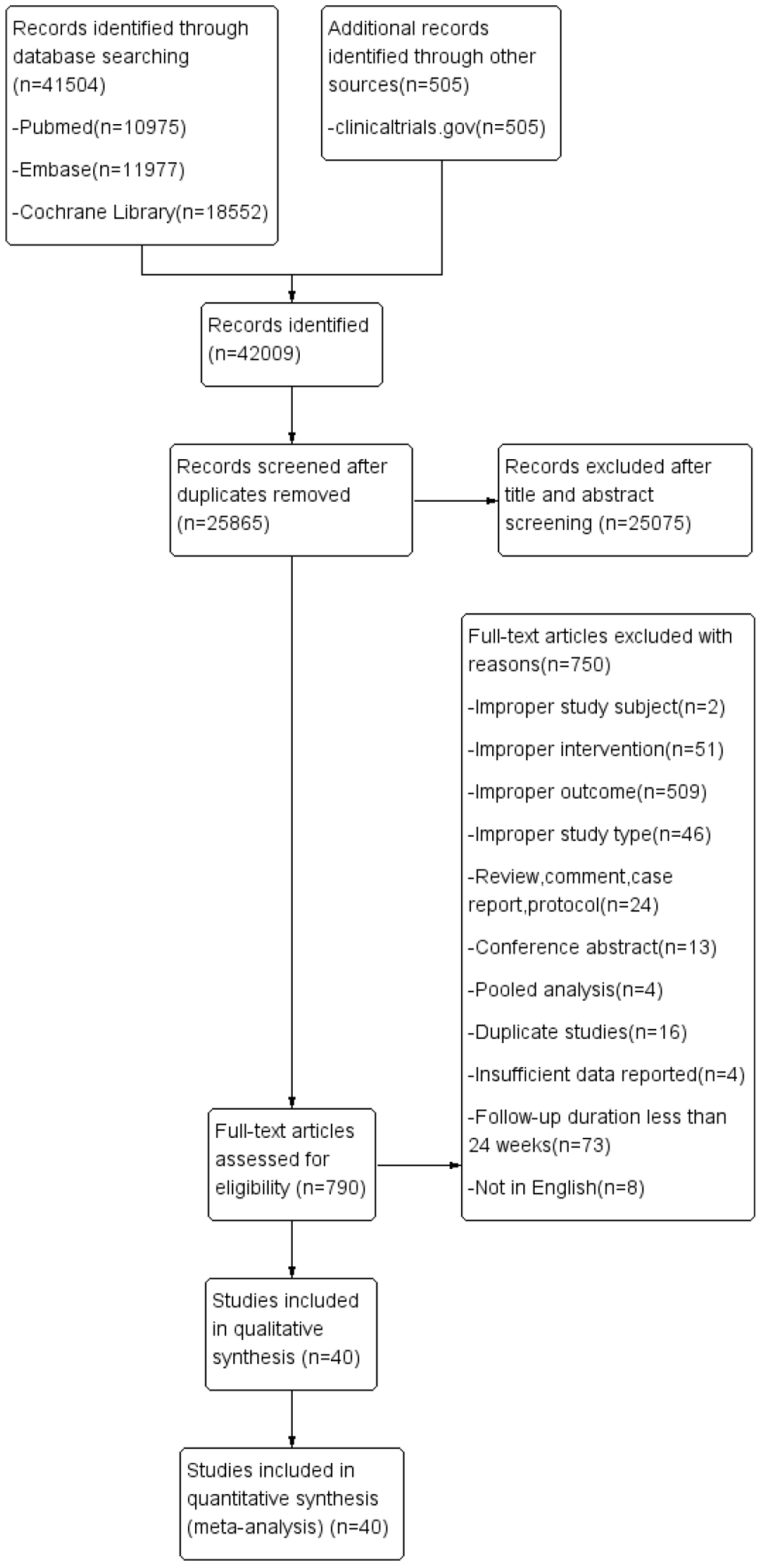
Flow diagram for study identification and inclusion.

This study retrieved a total of 42,009 articles based on the formulated search strategy. After excluding 16,144 duplications and further screening based on titles and abstracts, 25,075 articles were excluded, leaving 790 relevant studies. Following full-text review, an additional 750 articles were excluded, resulting in a final inclusion of 40 articles (7, 8, 14–16, 18, 22, 23, 27–58), comprising 42 studies and involving a total of 150,405 participants (Figure 1). Among these, there were 16 studies on statins (63,335 participants), 4 studies on ezetimibe (23,831 participants), and 24 studies on PCSK9 inhibitors (65,146 participants). Thirty-four studies reported neurocognitive events (141,431 participants), while 12 studies reported scores from neurocognitive assessment scales (59,299 participants). See Table 1 for basic characteristics of the included studies.

**Table 1:** Baseline characteristics of included studies.

Trial	Population	Mean age (years)	Intervention	Control	Background treatment	Cognitive outcome	Number	Follow-up (weeks)
Janik 2021 (22)	FH or patients with high cardiovascular risk	63.0	Alirocumab 75/150 mg q2w	Placebo	Maximally tolerated statin therapy	Adverse events + test scores	2,176	96
ODYSSEY CHOICE I 2016 (27)	Hypercholesterolemia with moderate or high cardiovascular risk	60.9	Alirocumab 300 mg q4w/150 mg q2w	Placebo	Statin or no statin	Adverse events	688	56
ODYSSEY CHOICE II 2016 (28)	Hypercholesterolemia	62.7	Alirocumab 75/150 mg q2w	Placebo	Standard therapy	Adverse events	174	32
ODYSSEY COMBO I 2015 (29)	Hypercholesterolemia and CHD or equivalent	63.0	Alirocumab 75/150 mg q2w	Placebo	Maximally tolerated statin therapy	Adverse events	316	52
ODYSSEY COMBO II 2017 (30)	Hypercholesterolemia and CHD or equivalent	61.6	Alirocumab 75 mg/150 mg q2w + oral placebo qd	Ezetimibe 10 mg qd + injection placebo q2w	Maximally tolerated statin therapy	Adverse events	720	112
ODYSSEY DM-DYSLIPIDEMIA 2018 (31)	T2DM with mixed hypercholesterolemia	63.2	Alirocumab 75/150 mg q2w	Standard therapy	Maximally tolerated statin therapy	Adverse events	413	24
ODYSSEY DM-INSULIN 2017 (32)	T1DM or T2DM with high cardiovascular risk	62.8	Alirocumab 75/150 mg q2w	Placebo	None	Adverse events	517	32
ODYSSEY EAST 2020 (33)	Hypercholesterolemia with high cardiovascular risk	58.6	Alirocumab 75 mg/150 mg q2w + oral placebo qd	Ezetimibe 10 mg qd + injection placebo q2w	Maximally tolerated statin therapy	Adverse events	615	32
ODYSSEY FH I 2015 (34)	FH	52.0	Alirocumab 75/150 mg q2w	Placebo	Maximally tolerated statin therapy	Adverse events	486	78
ODYSSEY FH II 2015 (34)	FH	53.2	Alirocumab 75/150 mg q2w	Placebo	Maximally tolerated statin therapy	Adverse events	249	78
ODYSSEY HIGH FH 2016 (35)	FH	50.6	Alirocumab 150 mg q2w	Placebo	Other cholesterol-lowering therapy	Adverse events	107	78
ODYSSEY KT 2018 (36)	Hypercholesterolemia with high cardiovascular risk	60.6	Alirocumab 75/150 mg q2w	Placebo	Maximally tolerated statin therapy	Adverse events	199	32
ODYSSEY LONG TERM 2015 (37)	Patients with high cardiovascular risk	60.5	Alirocumab 150 mg q2w	Placebo	Maximally tolerated statin therapy	Adverse events	2,341	86
ODYSSEY OPTIONS I 2015 (38)	Patients with high cardiovascular risk	64.0	Alirocumab 75/150 mg q2w + oral placebo qd	Ezetimibe 10 mg qd + injection placebo q2w	Atorvastatin 20 mg/40 mg qd	Adverse events	206	32
ODYSSEY OPTIONS II 2016 (39)	Hypercholesterolemia with high cardiovascular risk	60.9	Alirocumab 75/150 mg q2w + oral placebo qd	Ezetimibe 10 mg qd + injection placebo q2w	Rosuvastatin 10 mg/20 mg qd	Adverse events	204	32
ODYSSEY OUTCOMES 2018 (8)	ACS	58.6	Alirocumab 75 mg q2w	Placebo	High-intensity or maximally tolerated statin therapy	Adverse events	18,924	146
PACMAN-AMI 2022 (40)	AMI	58.5	Alirocumab 150 mg q2w	Placebo	Rosuvastatin 20 mg qd	Adverse events	300	52
FOURIER 2017 (41, 70)	ASCVD	62.5	Evolocumab 140 mg q2w/420 mg qm	Placebo	Statin	Adverse events + test scores	27,564	114
GLAGOV 2016 (42)	CHD	59.8	Evolocumab 420 mg qm	Placebo	Statin	Adverse events	970	76
HAUSER-RCT 2022 (43, 71)	Pediatric FH	13.7	Evolocumab 420 mg qm	Placebo	Other cholesterol-lowering therapy	Test scores	158	24
OSLER-1/2 2015 (18)	CHD or equivalent or hypercholesterolemia	57.9	Evolocumab 140 mg q2w/420 mg qm + standard therapy	Standard therapy	None	Adverse events	4,465	48
Kastelein 2016 (44)	Hypercholesterolemia	58.8	Frovocimab 300 mg q4w	Placebo q4w	Standard therapy	Adverse events	176	24
ORION-10 2020 (45)	ASCVD	66.1	Inclisiran 284 mg q6m	Placebo	Maximally tolerated statin therapy	Adverse events	1,561	77
ORION-11 2020 (45)	ASCVD or equivalent	64.8	Inclisiran 284 mg q6m	Placebo	Maximally tolerated statin therapy	Adverse events	1,617	77
CRISP 1997 (46)	Older adult with hypercholesterolemia	71.5	Lovastatin 40 mg qd	Placebo	None	Test scores	287	26
Muldoon 2000 (14)	Middle-aged and young patients with hypercholesterolemia	46.4	Lovastatin 20 mg qd	Placebo	None	Test scores	192	26
Carlsson 2002 (47)	Older adult with hypercholesterolemia	76.3	Pravastatin 40 mg qd	Tocopherol 400 IU qd + placebo	None	Test scores	41	26
PROSPER 2002 (15)	Older adult with a history of or risk factors for vascular disease	75.3	Pravastatin 40 mg qd	Placebo	None	Test scores	5,804	167
HPS 2002 (16, 72)	Patients coronary disease, other occlusive arterial disease, or diabetes	64.0	Simvastatin 40 mg qd	Placebo	None	Adverse events + test scores	20,536	260
Li 2017 (48)	Healthy persons	55.9	Simvastatin 40 mg qd	Placebo	None	Adverse events + test scores	49	52
STATCOPE 2014 (49)	COPD	62.3	Simvastatin 40 mg qd	Placebo	None	Adverse events	885	92
APPLE 2012 (50)	Pediatric SLE	15.7	Atorvastatin 10 mg/20 mg qd	Placebo	Standard therapy	Adverse events	221	156
ASCOT-LLA 2017 (51, 73)	Hypertension	63.1	Atorvastatin 10 mg qd	Placebo	None	Adverse events	10,350	172
Marlatt 2019 (52)	Young adult survivors of childhood acute lymphoblastic leukemia or non-Hodgkin’s lymphoma	26.8	Atorvastatin 40 mg qd	Placebo	None	Adverse events	27	26
Parale 2006 (23)	Cardiovascular disease or hypercholesterolemia	56.5	Atorvastatin 10 mg qd	Placebo	None	Test scores	97	52
AURORA 2009 (53)	Patients treated with hemodialysis	64.2	Rosuvastatin 10 mg qd	Placebo	None	Adverse events	2,776	166
HOPE-3 2019 (54)	Older adult with moderate cardiovascular risk	74.1	Rosuvastatin 10 mg qd	Placebo	None	Test scores	2,361	297
JUPITER 2008 (55)	Persons with high-sensitivity CRP	65.7	Rosuvastatin 20 mg qd	Placebo	None	Adverse events	17,802	99
SEAS 2008 (56)	Aortic stenosis	67.6	Ezetimibe 10 mg qd + simvastatin 40 mg qd	Placebo	None	Adverse events	1,873	227
Tendolkar 2012 (57)	Older adult, stroke-free patients with AF	74.0	Atorvastatin 10 mg/20 mg qd + ezetimibe 10 mg qd	Placebo	None	Test scores	34	52
IMPROVE-IT 2015 (7)	ACS	63.6	Simvastatin 40 mg qd + ezetimibe 10 mg qd	Simvastatin 40 mg qd + placebo	None	Adverse events	18,144	312
RACING 2022 (58)	ASCVD	64.0	Ezetimibe 10 mg qd + rosuvastatin 10 mg qd	Rosuvastatin 20 mg qd	None	Adverse events	3,780	156

FH, familial hypercholesterolemia; CHD, coronary heart disease; T2DM, type 2 diabetes mellitus; T1DM, type 1 diabetes mellitus; ACS, acute coronary syndrome; AMI, acute myocardial infarction; ASCVD, atherosclerotic cardiovascular disease; COPD, chronic obstructive pulmonary disease; SLE, systemic lupus erythematosus.

### Risk of bias

**Figure 2:**
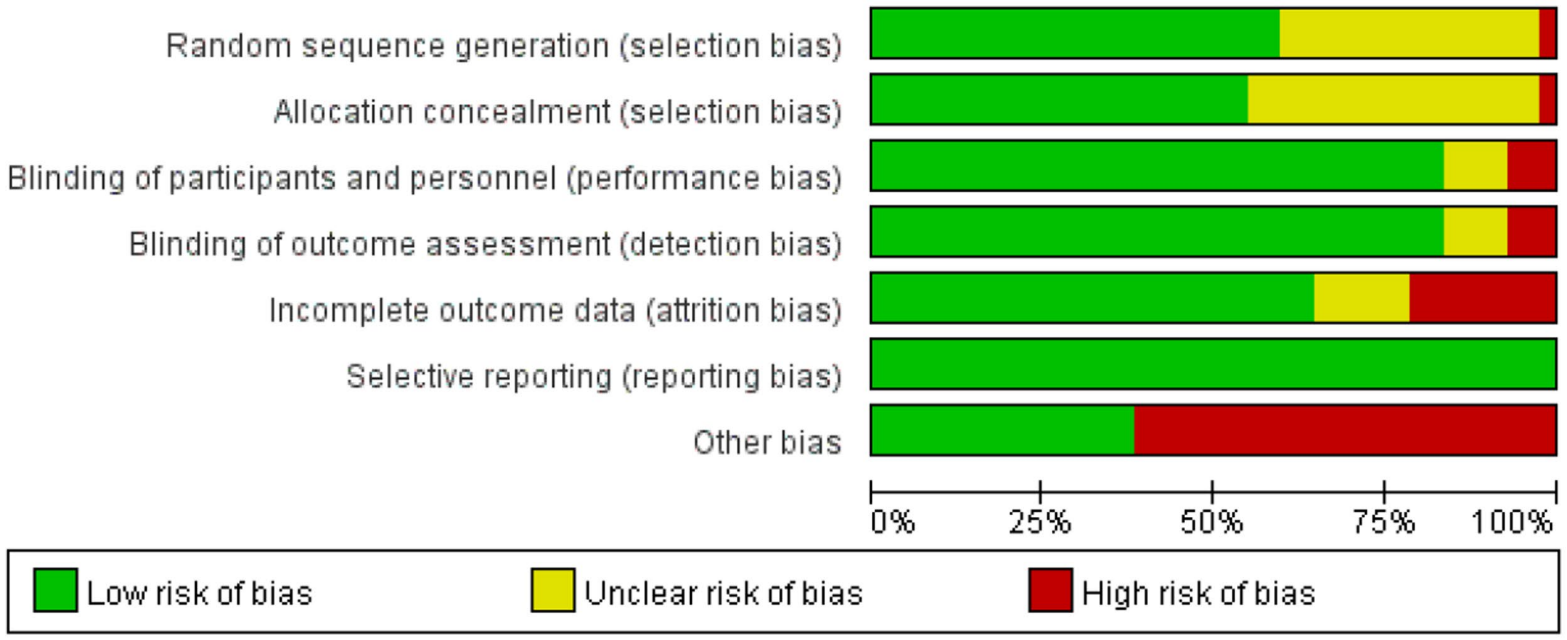
Risk of bias graph.

**Figure 3:**
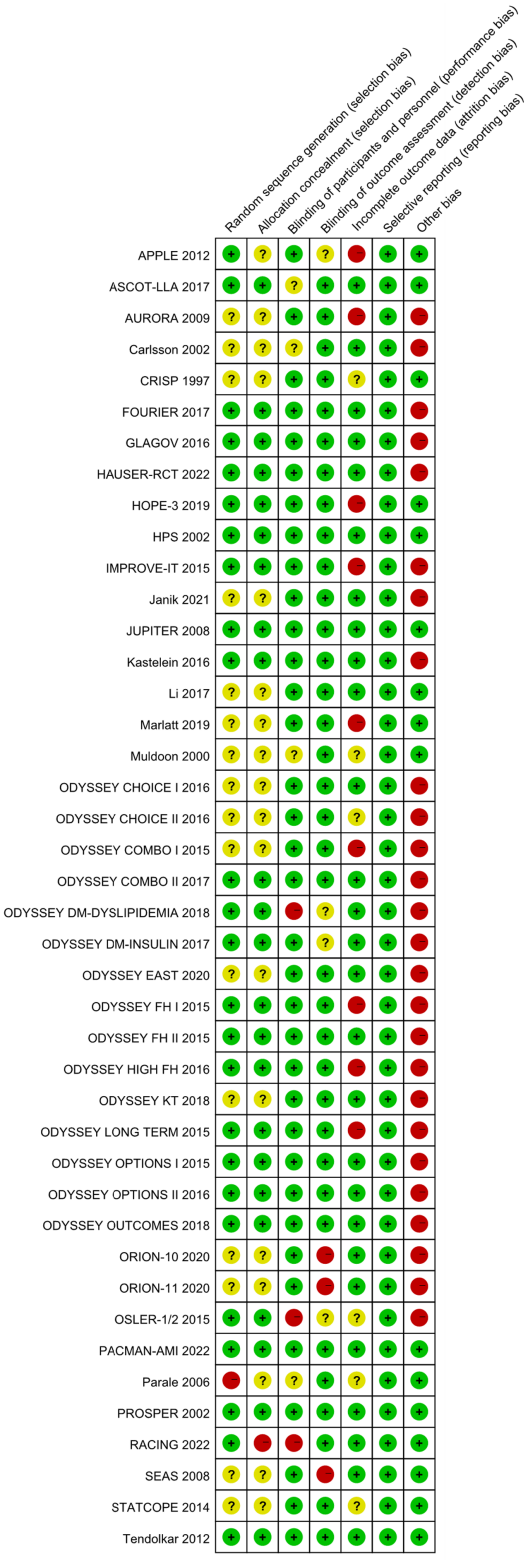
Risk of bias summary.

This study conducted bias risk assessments on the 42 included studies (see Figures 2, 3). Among them: 1 study grouped participants based on drug indications; 16 studies did not report specific methods for generating random sequences; 1 study did not conceal allocation; 16 studies did not report specific methods for allocation concealment; 3 studies were open-label in design; 3 studies did not blind outcome assessors; 9 studies had a dropout rate greater than 20%; 26 studies were sponsored by pharmaceutical companies, with involvement in the main part of the research.

### Incidence of neurocognitive events

**Figure 4:**
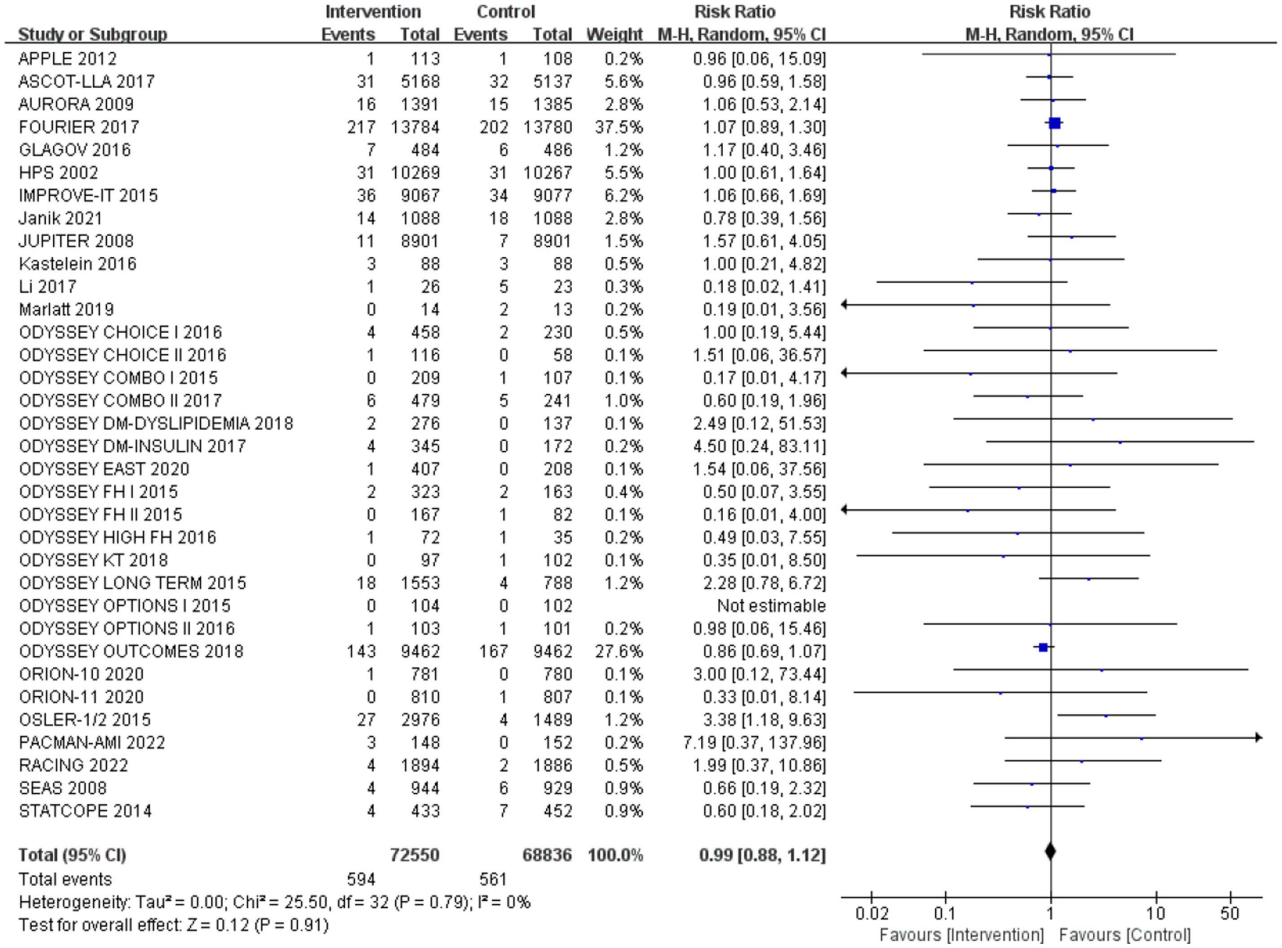
The effect of cholesterol-lowering drugs on neurocognitive events.

After analysing 34 studies that reported neurological cognitive events, the intervention group showed a lower risk of neurocognitive events compared to the control group, but the difference was not statistically significant (RR: 0.99, 95% CI: 0.88 to 1.12). No significant heterogeneity was observed (*Q*-test *p* = 0.79, *I*^2^ = 0%) (Figure 4).

**Figure 5:**
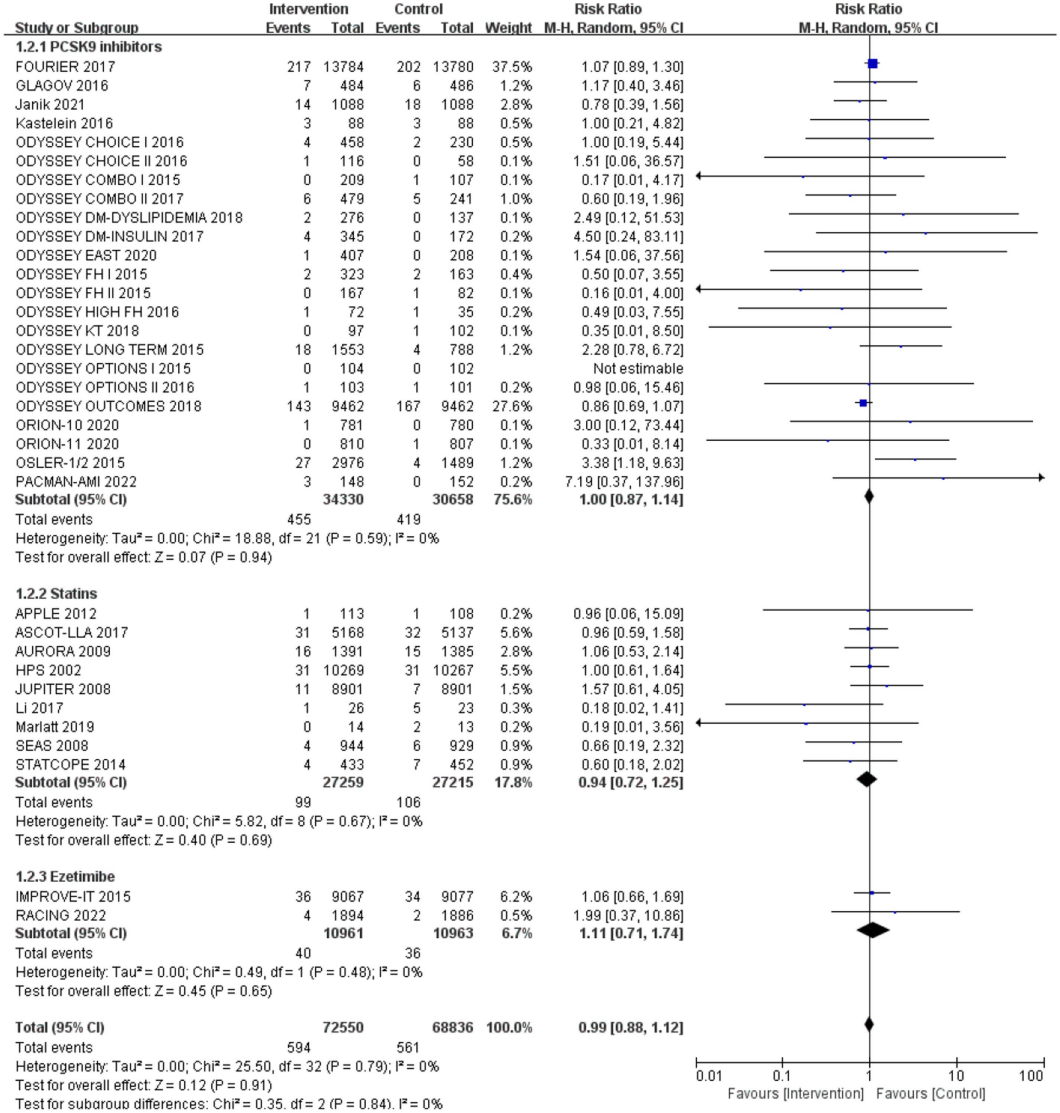
The effects of each cholesterol-lowering drug on neurocognitive events.

**Figure 6:**
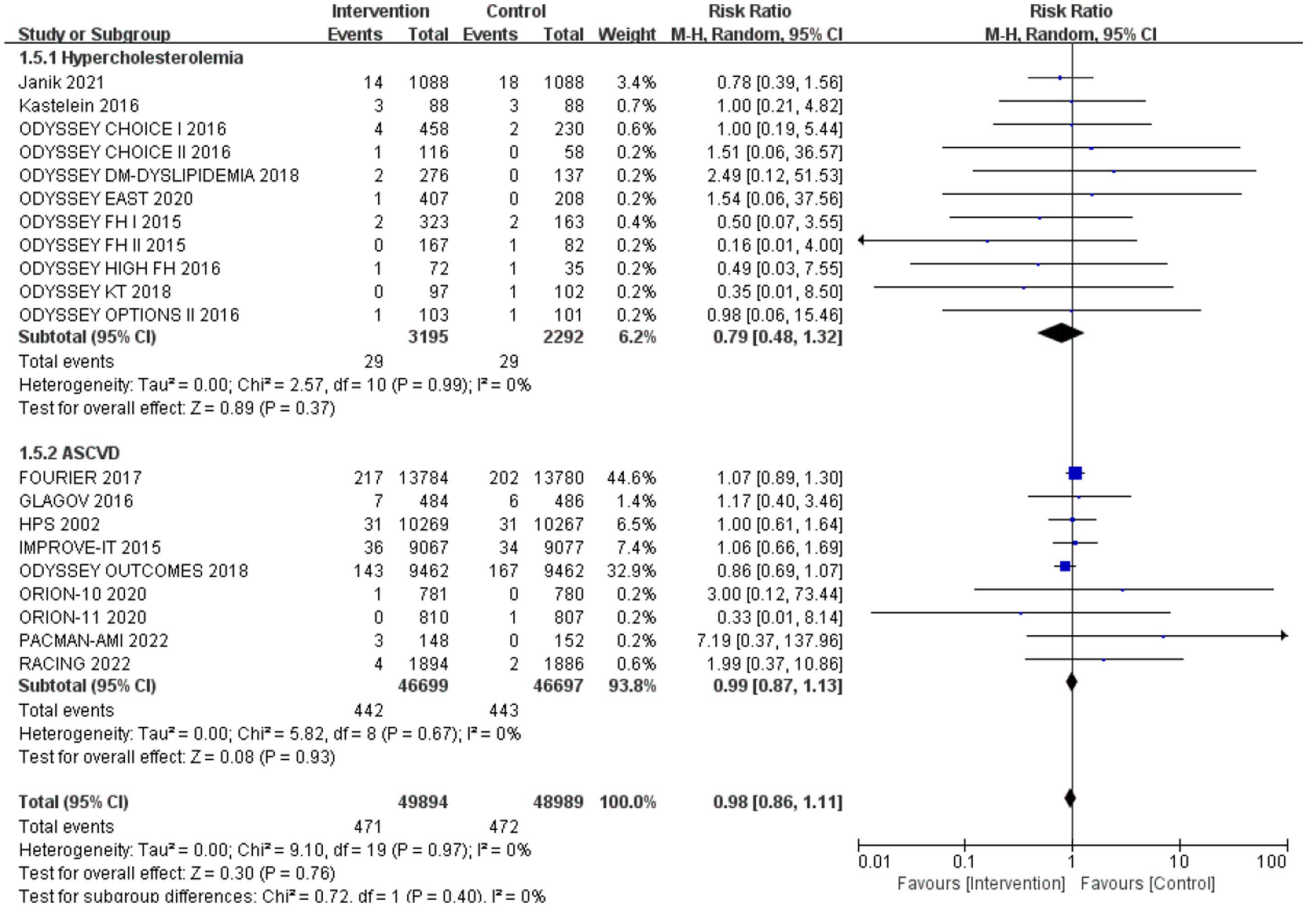
The effect of different participant disease profiles on neurocognitive events.

**Figure 7:**
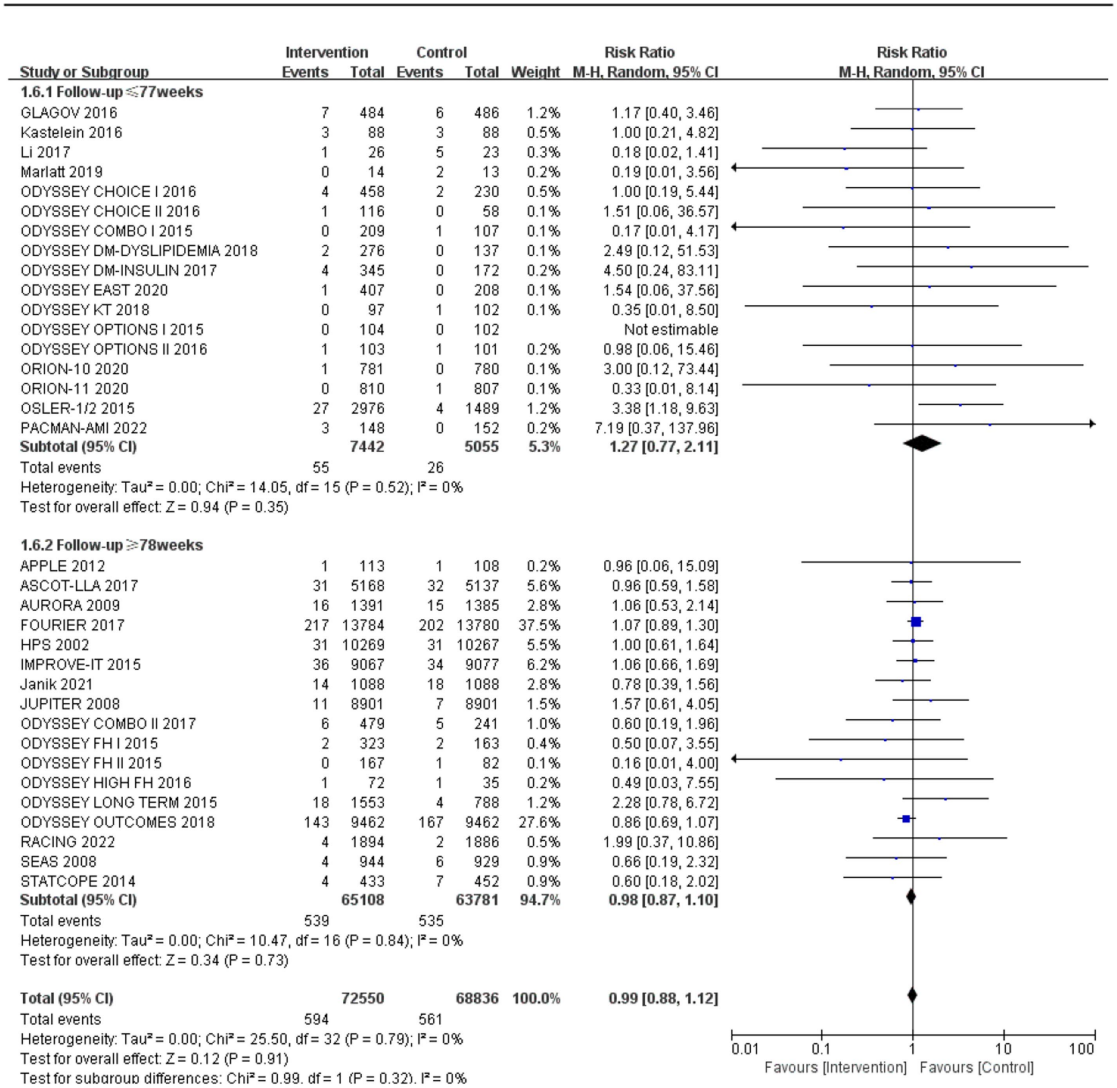
The effect of follow-up on neurocognitive events.

**Figure 8:**
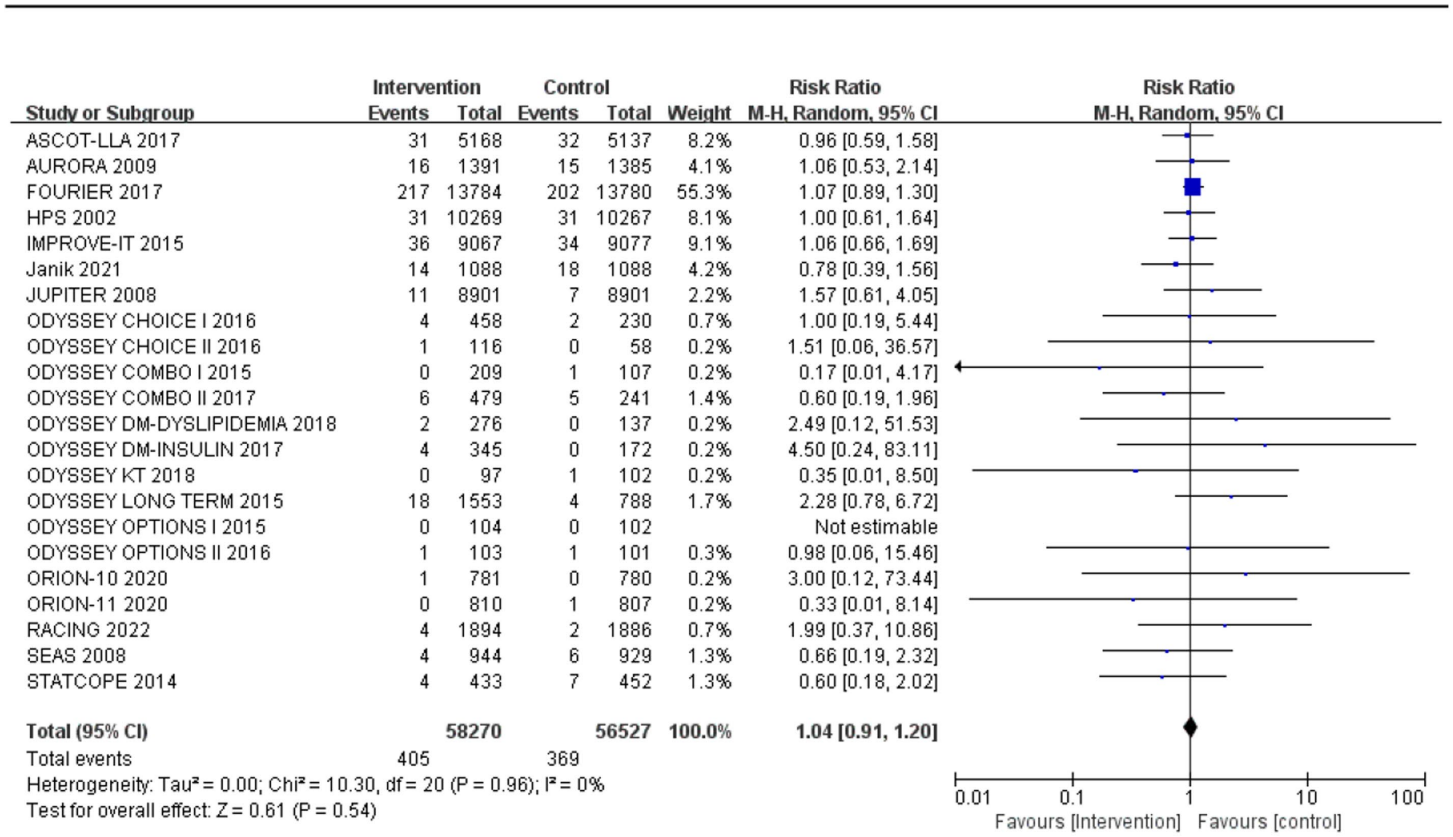
The incidence of neurocognitive events in studies with an average age over 60 years old.

**Figure 9:**
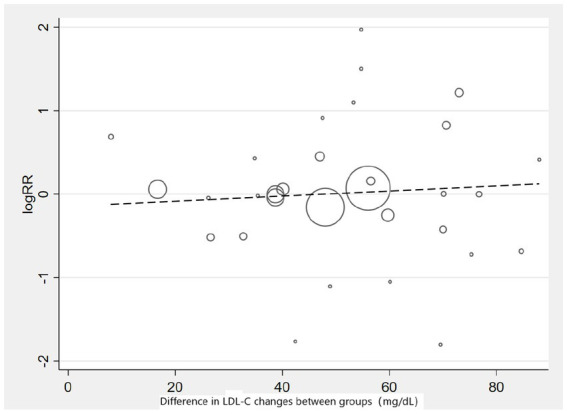
Meta-regression graph on the occurrence risk of neurocognitive events and absolute changes in LDL-C.

**Figure 10:**
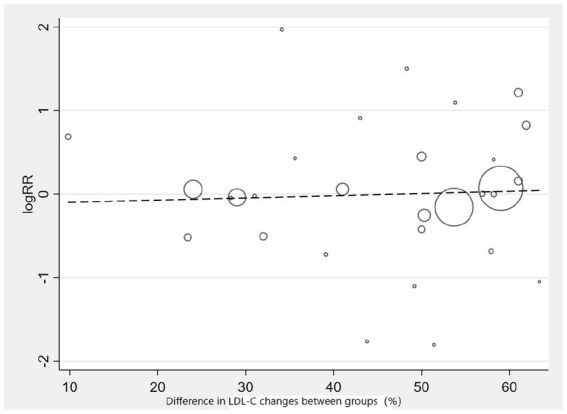
Meta-regression graph on the occurrence risk of neurocognitive events and percentage changes in LDL-C.

Subgroup analysis based on intervention measures (Figure 5) found that statins (RR: 0.94, 95% CI: 0.72 to 1.25), ezetimibe (RR: 1.11, 95% CI: 0.71 to 1.74), and PCSK9 inhibitors (RR: 1.00, 95% CI: 0.87 to 1.14) did not increase the risk of neurocognitive events, respectively. Subgroup analysis based on participant disease profiles (Figure 6) indicated that cholesterol-lowering therapy did not increase neurocognitive events in populations with hypercholesterolemia or atherosclerotic cardiovascular disease. When stratified by median follow-up 77.5 weeks (Figure 7), extended follow-up was not associated with elevated neurocognitive events. In the older adult subgroup, mean age ≥60 years (Figure 8), lipid-lowering drugs showed no adverse effect on neurocognitive function (RR: 1.04, 95% CI: 0.91 to 1.20). Meta-regression analyses indicated that the risk of neurocognitive events did not change with absolute changes in LDL-C before and after intervention (*β*: 0.0031, 95% CI: −0.0071 to 0.0132), nor with percentage changes in LDL-C (*β*: 0.0026, 95% CI: −0.0090 to 0.0143) (Figures 9, 10).

### Scores on neurocognitive assessment scales

Twelve studies reported scores of neurocognitive test scales. We categorized neurocognitive scales into six domains: attention, psychomotor speed, executive function, working memory, memory, and global cognition (Table 2).

**Table 2:** Results of neurocognitive test scales.

Cognitive domains	Number	Effect size (SMD and 95% CI)
Attention	4	0.18 (−0.33, 0.70)
Psychomotor speed	10	0.06 (−0.05, 0.18)
Executive function	5	−0.01 (−0.06, 0.04)
Working memory	4	0.18 (−0.25, 0.61)
Memory	7	0.00 (−0.03, 0.04)
Global cognition	4	0.01 (−0.02, 0.03)

#### The effect of cholesterol-lowering drugs on attention

**Figure 11:**
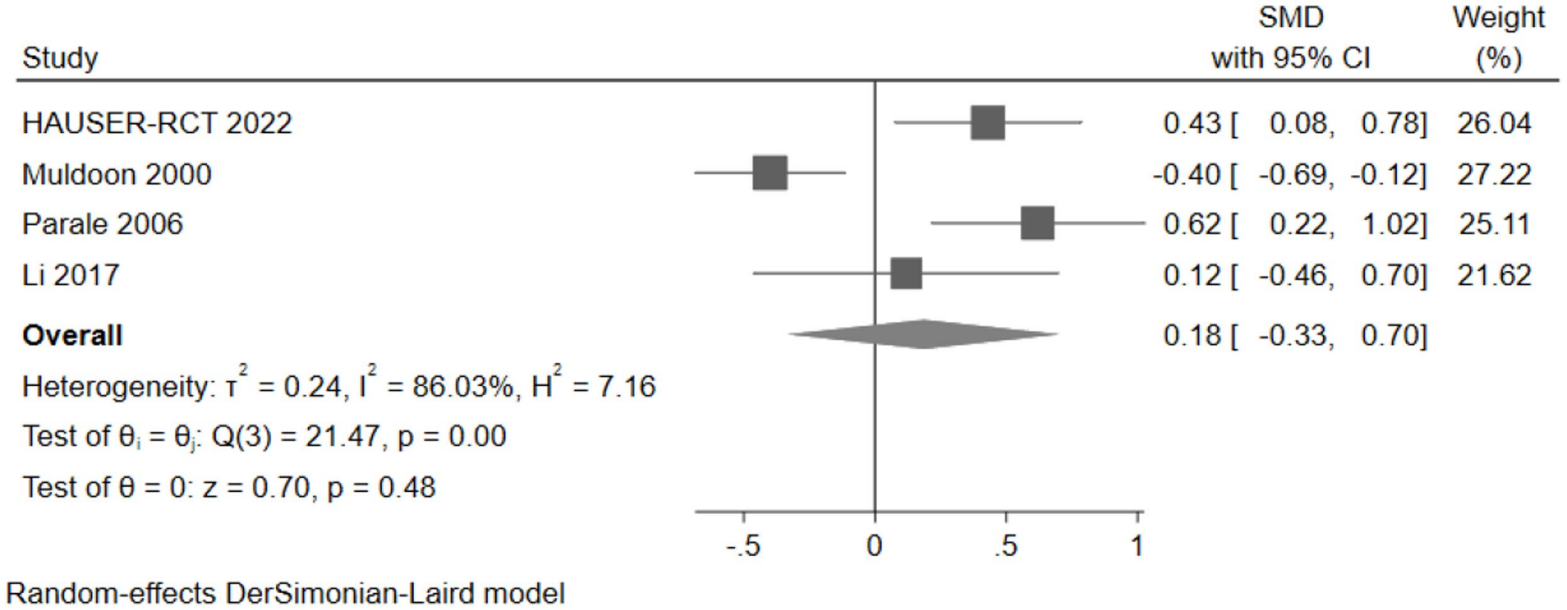
The effect of cholesterol-lowering drugs on attention.

Four studies (477 participants) assessed attention, with the pooled analysis showing no significant changes in attention for intervention group participants compared to controls (SMD: 0.18, 95% CI: −0.33 to 0.70) (Figure 11).

#### The effect of cholesterol-lowering drugs on psychomotor speed

**Figure 12:**
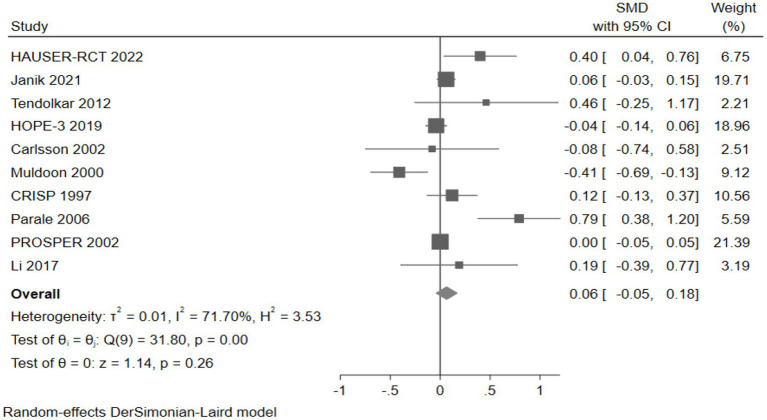
The effect of cholesterol-lowering drugs on psychomotor speed.

Ten studies (10,295 participants) assessed psychomotor speed, with pooled analysis showing no significant changes in psychomotor speed for intervention group participants compared to controls (SMD: 0.06, 95% CI: −0.05 to 0.18) (Figure 12).

#### The effect of cholesterol-lowering drugs on executive function

**Figure 13:**
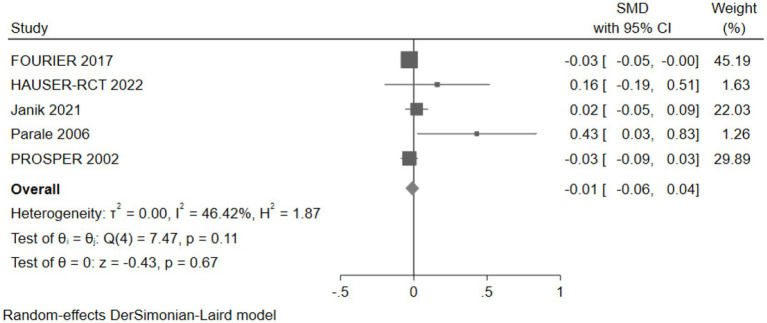
The effect of cholesterol-lowering drugs on executive function.

Five studies (30,733 participants) assessed executive function, with pooled analysis showing no significant changes in executive function for intervention group participants compared to controls (SMD: -0.01, 95% CI: −0.06 to 0.04) (Figure 13).

#### The effect of cholesterol-lowering drugs on working memory

**Figure 14:**
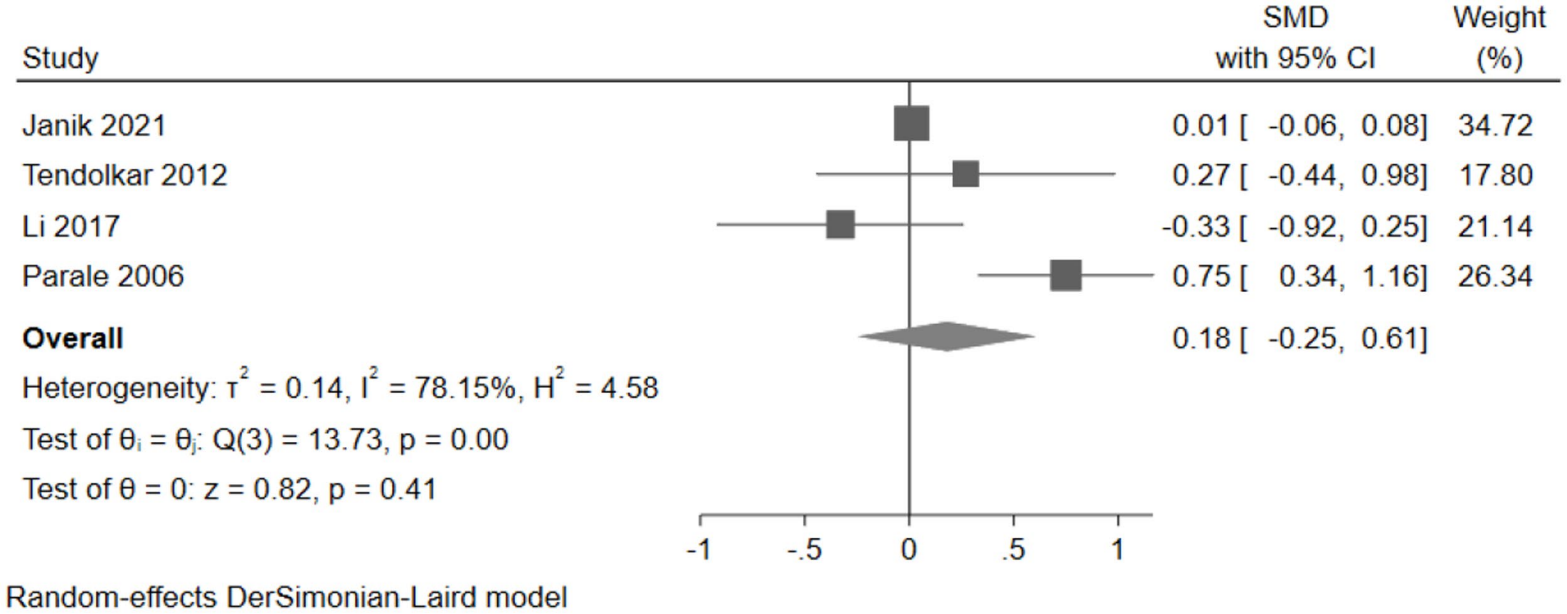
The effect of cholesterol-lowering drugs on working memory.

Four studies (2,260 participants) assessed working memory, with pooled analysis showing no significant changes in working memory for intervention group participants compared to controls (SMD: 0.18, 95% CI: −0.25 to 0.61) (Figure 14).

#### The effect of cholesterol-lowering drugs on memory

**Figure 15:**
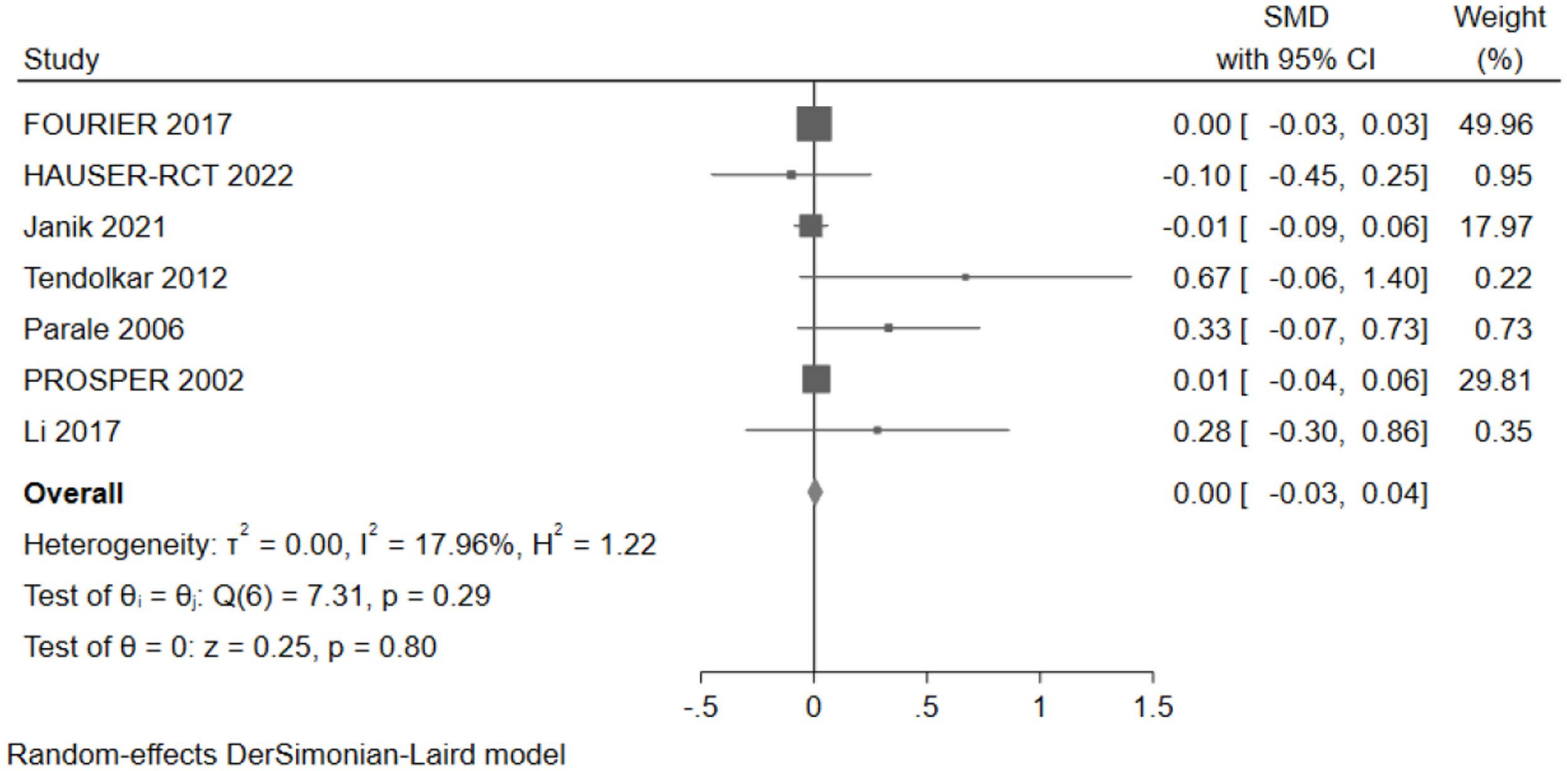
The effect of cholesterol-lowering drugs on memory.

Seven studies (30,814 participants) assessed memory, with pooled analysis showing no significant changes in memory for intervention group participants compared to controls (SMD: 0.00, 95% CI: −0.03 to 0.04) (Figure 15).

#### The effect of cholesterol-lowering drugs on global cognition

**Figure 16:**
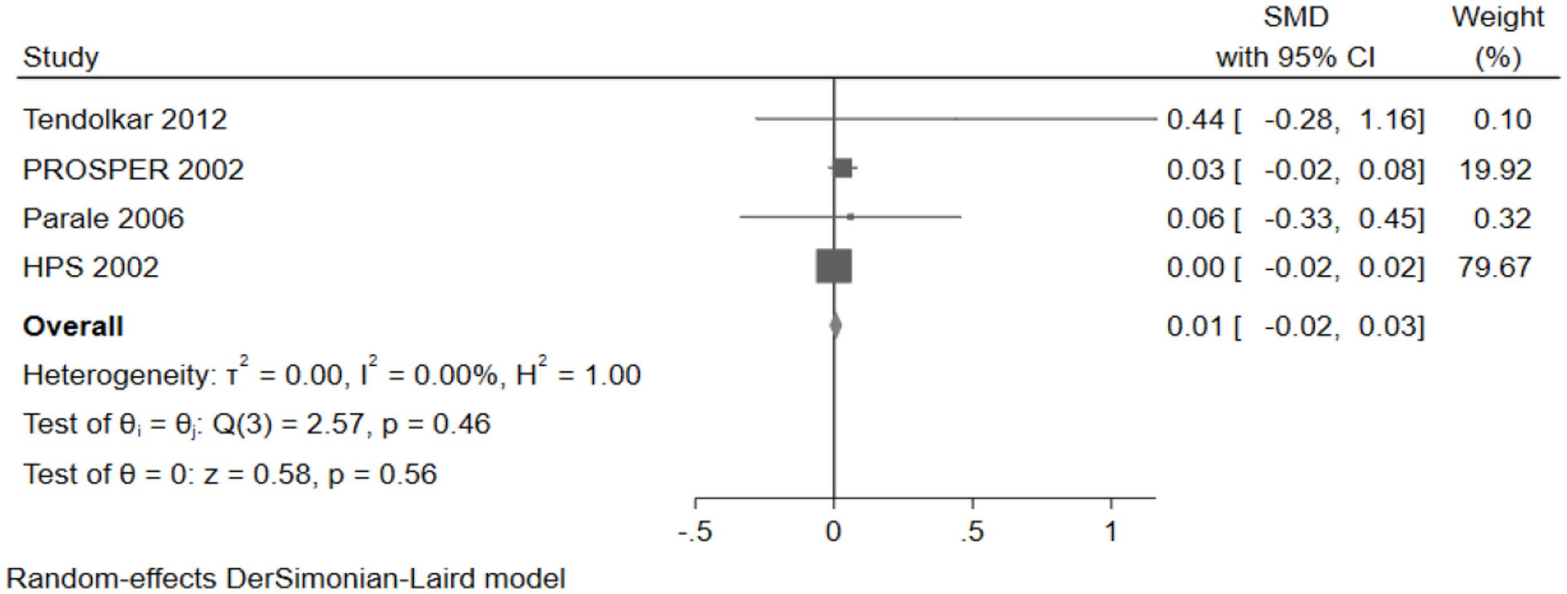
The effect of cholesterol-lowering drugs on global cognition.

Four studies (26,468 participants) assessed global cognition, with pooled analysis showing no significant changes in global cognition for intervention group participants compared to controls (SMD: 0.01, 95% CI: −0.02 to 0.03) (Figure 16).

In summary, across various domains of neurocognitive function, we found no significant impact of cholesterol-lowering medications.

### Sensitivity analysis

**Figure 17:**
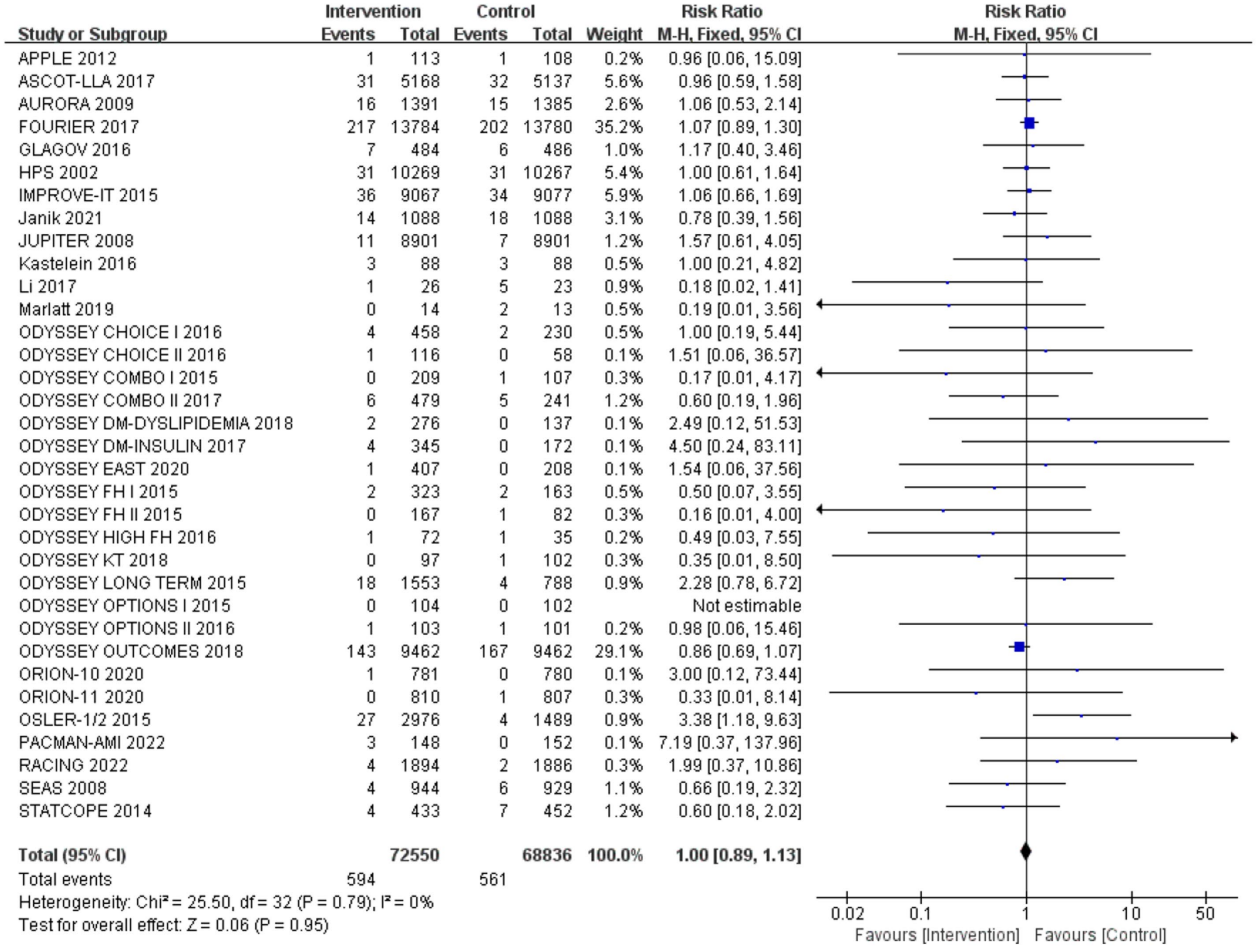
Sensitivity analysis: change the effect model.

**Figure 18:**
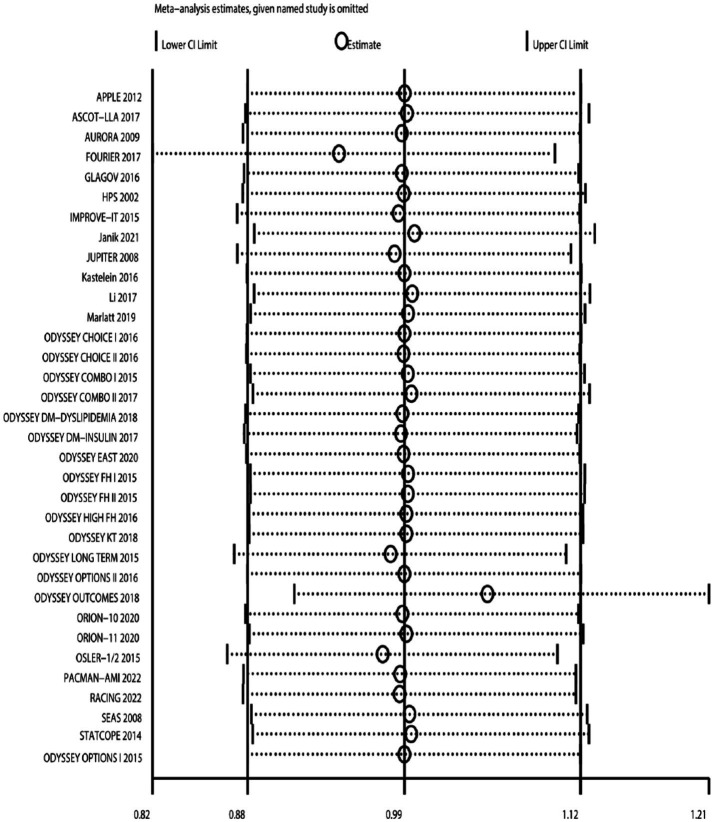
Sensitivity analysis: sequentially excluding literature.

After changing the effect model, there was no significant change in the incidence rate of neurocognitive events (Figure 17). Sensitivity analysis was also conducted by sequentially excluding studies, showing that after excluding individual studies, the incidence rate of neurocognitive events did not significantly change compared to the overall rate (RR fluctuated between 0.94 and 1.05) (Figure 18). Sensitivity analysis indicated robustness of the meta-analysis results.

### Publication bias

**Figure 19:**
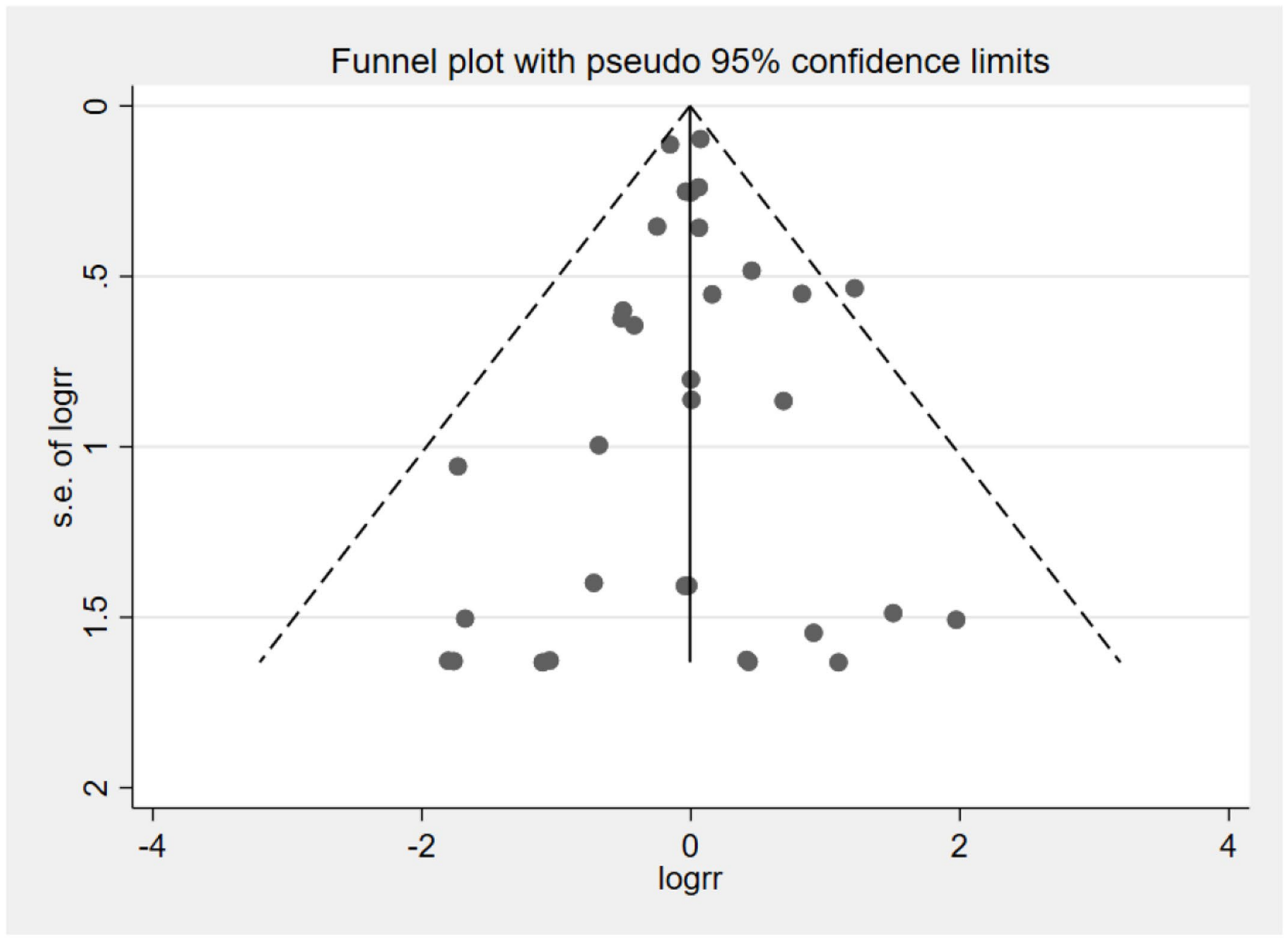
Funnel plot of neurocognitive event studies.

The funnel plot was used to assess publication bias in the outcome of neurocognitive events (Figure 19). The result suggested that studies were symmetrically distributed in the funnel plot. Combining Begg’s test (*Z* = 0.64, *p* = 0.525) and Egger’s test (*t* = −0.15, *p* = 0.882) results, we concluded that there was no publication bias in the included studies.

## Discussion

Some perspectives suggest that cholesterol, as a critical component of the brain, may play a role in neurological function, and its reduction could potentially lead to dysfunction. Firstly, lowering LDL-C levels may interfere with neurotransmitter synthesis. The molar ratio of cholesterol to phospholipids in cell membranes has a significant impact on membrane lipid microviscosity; a lower cholesterol content decreases membrane viscosity, thereby increasing membrane fluidity and reducing the exposure of membrane proteins to the extracellular environment. This alteration may impact various membrane protein functions, such as transport mechanisms, signal transduction, receptor binding, enzymatic activity, and protein phosphorylation, thereby potentially affecting neurotransmitter synthesis as well as synaptic neurotransmitter uptake and binding (59). Furthermore, lowering LDL-C levels may also impact the function of myelin sheaths. Unlike other cellular membranes, myelin sheaths consist of approximately 70% lipids and 30% proteins, with cholesterol accounting for about 40% of the total lipid content. These sheaths envelop axons and form periodic gaps known as nodes of Ranvier, which facilitate saltatory conduction of action potentials. A decrease in cholesterol levels may compromise the structural integrity of myelin sheaths, potentially resulting in delayed conduction of action potentials (60).

However, one study has investigated plasma LDL-C levels in full-term newborns, reporting an average level of approximately 20 mg/dL, which suggests that humans are capable of maintaining essential physiological functions at such low plasma LDL-C concentrations (61). Furthermore, LDL receptors achieve half-maximal binding at plasma LDL-C concentrations of approximately 2.5 mg/dL, and the plasma LDL-C level is about fivefold higher than that in the interstitial fluid. Therefore, a theoretical plasma LDL-C concentration of 12.5 mg/dL would be sufficient to ensure adequate cellular uptake of cholesterol (62). Additionally, evidence suggests that brain cholesterol content is independent of the peripheral circulation system due to the presence of the blood–brain barrier. The brain synthesizes cholesterol autonomously and does not rely on dietary or hepatic sources for its structural integrity or functional performance (63). Moreover, lipidomic studies highlight a key mechanistic insight: lipid-lowering therapies can alter circulating lipoprotein patterns without disrupting cholesterol homeostasis in the brain. The intact blood–brain barrier and autonomous regulatory mechanisms appear to safeguard against potential depletion from systemic LDL-C reduction. Furthermore, it is found that inclisiran can improve lipid profile and pulse wave velocity in familial hypercholesterolemia subjects (64). It means cholesterol-lowering therapies have been associated with improvements in arterial stiffness and vascular function. Cholesterol-lowering medications have been shown to reduce the incidence of ASCVD events, thereby potentially lowering the risk of vascular dementia.

Currently, evidence concerning a potential increased risk of neurocognitive events associated with cholesterol-lowering medications primarily derives from studies involving statins, often based on case reports (65). Conversely, our study exclusively includes randomized controlled trials on statins, thereby providing higher-quality evidence compared to case reports. None of the studies included in our analysis reported any adverse effects on neurocognitive function associated with statin use, even for lipophilic statins such as simvastatin and atorvastatin, which have a greater propensity to cross the blood–brain barrier (16, 51).

In the studies included in our analysis, research on cholesterol absorption inhibitors remains limited. One potential explanation is that these agents exert only a modest effect on cholesterol reduction, as the majority of cholesterol in the body is endogenously synthesized, with only a small fraction being absorbed from the intestines. For instance, monotherapy with ezetimibe 10 mg/day reduces LDL-C levels by approximately 18.6% (66). Furthermore, there is currently no conclusive evidence indicating that cholesterol absorption inhibitors possess pharmacological properties that may lead to impairments in neurocognitive function.

In studies evaluating the effects of PCSK9 inhibitors on neurocognitive function, the OSLER study (18) indicated a potential increase in the risk of neurocognitive events associated with PCSK9 inhibitor use (relative risk: 3.38, 95% CI: 1.18–9.63). Study participants had previously been enrolled in phase II or III clinical trials of evolocumab and were predominantly individuals with coronary artery disease or hypercholesterolemia. Participants were randomized into two groups based on whether evolocumab was added to standard therapy, with a median follow-up duration of 11.1 months. Although the safety analysis revealed a higher incidence of neurocognitive events in the intervention group (0.9%) compared to the control group (0.3%), it is important to note that this was an open-label study without blinding, and adverse events were self-reported, which may have led to an overestimation of the effect size due to nocebo effects. However, a systematic review of all available PCSK9 inhibitor trials found no evidence of impaired neurocognitive function (8, 18, 22, 27–45).

In the population utilizing cholesterol-lowering medications, a substantial proportion consists of older adult individuals, who are at increased risk for neurocognitive impairment. Among studies reporting neurocognitive events, those specifically included populations with a mean age exceeding 60 years did not demonstrate a significant increase in such events. However, none of these studies were specifically designed to focus on older adult populations. Among studies reporting neurocognitive test scores, five specifically enrolled older adult participants (15, 46, 47, 54, 57). Across all cognitive domains evaluated, these studies did not identify any evidence indicating that cholesterol-lowering medications adversely affect neurocognitive function in older adult individuals.

Our findings are generally consistent with those of previous meta-analyses (67, 68). Although one meta-analysis reported that PCSK9 inhibitor users exhibited an increased likelihood of experiencing neurocognitive events (odds ratio: 2.34, 95% CI: 1.11–4.93), this analysis included only six randomized controlled trials, which had a smaller combined sample size compared to the current study (69). Notably, two studies—ODYSSEY LONG TERM and OSLER2—accounted for 89.3% of the weight in this analysis, introducing potential selection bias (18, 37).

This study included a total of 42 randomized controlled trials, of which 34 reported outcomes related to neurocognitive events. No evidence was found to suggest that LDL-C reduction leads to an increased incidence of such events. Meta-regression analyses further indicated no dose–response relationship between either the absolute or relative reduction in LDL-C levels and the risk of neurocognitive events. Similarly, an analysis of 12 studies reporting neurocognitive test scores revealed no significant changes across five cognitive domains—attention, psychomotor speed, executive function, working memory, and memory—or in global cognitive function associated with the use of cholesterol-lowering medications. Therefore, we conclude that cholesterol-lowering drugs do not have adverse effects on neurocognitive function.

### Limitations

This study has several limitations. First, the study population was restricted to individuals with normal neurocognitive function, excluding those with pre-existing neurocognitive impairments. Second, some of the original studies were not specifically designed to assess neurocognitive function, and the reporting of some events was subjective, which may have introduced potential inaccuracies in the evaluation of neurocognitive outcomes. Meanwhile, only a limited number of studies included formal cognitive testing, and there was heterogeneity among the measurement tools used. Some tools detected subtle changes poorly. Moreover, several studies had a relatively short follow-up duration, with 13 studies reporting follow-up periods of only approximately six months. Additionally, evidence remains limited for individuals aged 75 years and older, underscoring the need for further research on the safety of cholesterol-lowering therapies in this older older adult population.

## Conclusion

Our study indicates that cholesterol-lowering agents, including statins, cholesterol absorption inhibitors, and PCSK9 inhibitors, do not have adverse effects on neurocognitive function. Reduction in LDL-C levels is not associated with a decline in neurocognitive performance. Furthermore, cholesterol-lowering therapy does not appear to impair neurocognitive function in individuals aged 60 years and older.

## Data Availability

The raw data supporting the conclusions of this article will be made available by the authors, without undue reservation.

## References

[1] Zhao D Liu J Wang M Zhang X Zhou M. Epidemiology of cardiovascular disease in China: current features and implications. Nat Rev Cardiol. (2019) 16:203–12. doi: 10.1038/s41569-018-0119-4, 30467329

[2] Ference BA Ginsberg HN Graham I Ray KK Packard CJ Bruckert E . Low-density lipoproteins cause atherosclerotic cardiovascular disease. 1. Evidence from genetic, epidemiologic, and clinical studies. A consensus statement from the European Atherosclerosis Society Consensus Panel. Eur Heart J. (2017) 38:2459–72. doi: 10.1093/eurheartj/ehx144, 28444290 PMC5837225

[3] Sirtori CR. The pharmacology of statins. Pharmacol Res. (2014) 88:3–11. doi: 10.1016/j.phrs.2014.03.002, 24657242

[4] Betters JL Yu L. NPC1L1 and cholesterol transport. FEBS Lett. (2010) 584:2740–7. doi: 10.1016/j.febslet.2010.03.030, 20307540 PMC2909875

[5] Pärn A Olsen D Tuvikene J Kaas M Borisova E Bilgin M . PCSK9 deficiency alters brain lipid composition without affecting brain development and function. Front Mol Neurosci. (2023) 15:1084633. doi: 10.3389/fnmol.2022.1084633, 36733269 PMC9887304

[6] Scandinavian Simvastatin Survival Study Group. Randomised trial of cholesterol lowering in 4444 patients with coronary heart disease: the Scandinavian Simvastatin Survival Study (4S). Lancet. (1994) 344:1383–9. 7968073

[7] Cannon CP Blazing MA Giugliano RP McCagg A White JA Theroux P . Ezetimibe added to statin therapy after acute coronary syndromes. N Engl J Med. (2015) 372:2387–97. doi: 10.1056/NEJMoa1410489, 26039521

[8] Schwartz GG Steg PG Szarek M Bhatt DL Bittner VA Diaz R . Alirocumab and cardiovascular outcomes after acute coronary syndrome. N Engl J Med. (2018) 379:2097–107. doi: 10.1056/NEJMoa1801174, 30403574

[9] Björkhem I Meaney S. Brain cholesterol: long secret life behind a barrier. Arterioscler Thromb Vasc Biol. (2004) 24:806–15. doi: 10.1161/01.atv.0000120374.59826.1b, 14764421

[10] Yuet WC Ebert D Jann M. Neurocognitive effects associated with proprotein convertase subtilisin-kexin type 9 inhibitor use: a narrative review. Ther Adv Drug Saf. (2021) 12:2042098620959271. doi: 10.1177/2042098620959271, 33763200 PMC7944525

[11] King DS Wilburn AJ Wofford MR Harrell TK Lindley BJ Jones DW. Cognitive impairment associated with atorvastatin and simvastatin. Pharmacotherapy. (2003) 23:1663–7. doi: 10.1592/phco.23.15.1663.31953, 14695047

[12] Evans MA Golomb BA. Statin-associated adverse cognitive effects: survey results from 171 patients. Pharmacotherapy. (2009) 29:800–11. doi: 10.1592/phco.29.7.800, 19558254

[13] Muldoon MF Ryan CM Sereika SM Flory JD Manuck SB. Randomized trial of the effects of simvastatin on cognitive functioning in hypercholesterolemic adults. Am J Med. (2004) 117:823–9. doi: 10.1016/j.amjmed.2004.07.041, 15589485

[14] Muldoon MF Barger SD Ryan CM Flory JD Lehoczky JP Matthews KA . Effects of lovastatin on cognitive function and psychological well-being. Am J Med. (2000) 108:538–46. doi: 10.1016/s0002-9343(00)00353-3, 10806282

[15] Shepherd J Blauw GJ Murphy MB Bollen EL Buckley BM Cobbe SM . Pravastatin in elderly individuals at risk of vascular disease (PROSPER): a randomised controlled trial. Lancet. (2002) 360:1623–30. doi: 10.1016/s0140-6736(02)11600-x, 12457784

[16] Heart Protection Study Collaborative Group. MRC/BHF heart protection study of cholesterol lowering with simvastatin in 20,536 high-risk individuals: a randomised placebo-controlled trial. Lancet. (2002) 360:7–22. doi: 10.1016/S0140-6736(02)09327-3 12114036

[17] Mach F Baigent C Catapano AL Koskinas KC Casula M Badimon L . 2019 ESC/EAS guidelines for the management of dyslipidaemias: lipid modification to reduce cardiovascular risk. Eur Heart J. (2020) 41:111–88. doi: 10.1093/eurheartj/ehz455, 31504418

[18] Sabatine MS Giugliano RP Wiviott SD Raal FJ Blom DJ Robinson J . Efficacy and safety of evolocumab in reducing lipids and cardiovascular events. N Engl J Med. (2015) 372:1500–9. doi: 10.1056/NEJMoa1500858, 25773607

[19] Moher D Liberati A Tetzlaff J Altman DG PRISMA Group. Preferred reporting items for systematic reviews and meta-analyses: the PRISMA statement. PLoS Med. (2009) 6:e1000097. doi: 10.1371/journal.pmed.1000097, 19621072 PMC2707599

[20] Harvey PD Sabbagh MN Harrison JE Ginsberg HN Chapman MJ Manvelian G . No evidence of neurocognitive adverse events associated with alirocumab treatment in 3340 patients from 14 randomized phase 2 and 3 controlled trials: a meta-analysis of individual patient data. Eur Heart J. (2018) 39:374–81. doi: 10.1093/eurheartj/ehx661, 29186504 PMC5837381

[21] Ying H Wang J Shen Z Wang M Zhou B. Impact of lowering low-density lipoprotein cholesterol with contemporary lipid-lowering medicines on cognitive function: a systematic review and meta-analysis. Cardiovasc Drugs Ther. (2021) 35:153–66. doi: 10.1007/s10557-020-07045-2, 32770521

[22] Janik MJ Urbach DV van Nieuwenhuizen E Zhao J Yellin O Baccara-Dinet MT . Alirocumab treatment and neurocognitive function according to the CANTAB scale in patients at increased cardiovascular risk: a prospective, randomized, placebo-controlled study. Atherosclerosis. (2021) 331:20–7. doi: 10.1016/j.atherosclerosis.2021.06.913, 34303265

[23] Parale GP Baheti NN Kulkarni PM Panchal NV. Effects of atorvastatin on higher functions. Eur J Clin Pharmacol. (2006) 62:259–65. doi: 10.1007/s00228-005-0073-z, 16489473

[24] Richardson K Schoen M French B Umscheid CA Mitchell MD Arnold SE . Statins and cognitive function: a systematic review. Ann Intern Med. (2013) 159:688–97. doi: 10.7326/0003-4819-159-10-201311190-00007, 24247674

[25] Higgins JP Altman DG Gøtzsche PC Jüni P Moher D Oxman AD . The cochrane collaboration's tool for assessing risk of bias in randomised trials. BMJ. (2011) 343:d5928. doi: 10.1136/bmj.d5928 22008217 PMC3196245

[26] Wan X Wang W Liu J Tong T. Estimating the sample mean and standard deviation from the sample size, median, range and/or interquartile range. BMC Med Res Methodol. (2014) 14:135. doi: 10.1186/1471-2288-14-135, 25524443 PMC4383202

[27] Roth EM Moriarty PM Bergeron J Langslet G Manvelian G Zhao J . A phase III randomized trial evaluating alirocumab 300 mg every 4 weeks as monotherapy or add-on to statin: ODYSSEY CHOICE I. Atherosclerosis. (2016) 254:254–62. doi: 10.1016/j.atherosclerosis.2016.08.043, 27639753

[28] Stroes E Guyton JR Lepor N Civeira F Gaudet D Watts GF . Efficacy and safety of alirocumab 150 mg every 4 weeks in patients with hypercholesterolemia not on statin therapy: the ODYSSEY CHOICE II study. J Am Heart Assoc. (2016) 5:e003421. doi: 10.1161/JAHA.116.003421, 27625344 PMC5079013

[29] Kereiakes DJ Robinson JG Cannon CP Lorenzato C Pordy R Chaudhari U . Efficacy and safety of the proprotein convertase subtilisin/kexin type 9 inhibitor alirocumab among high cardiovascular risk patients on maximally tolerated statin therapy: the ODYSSEY COMBO I study. Am Heart J. (2015) 169:906–15. doi: 10.1016/j.ahj.2015.03.004 26027630

[30] El Shahawy M Cannon CP Blom DJ McKenney JM Cariou B Lecorps G . Efficacy and safety of alirocumab versus ezetimibe over 2 years (from ODYSSEY COMBO II). Am J Cardiol. (2017) 120:931–9. doi: 10.1016/j.amjcard.2017.06.023, 28750828

[31] Ray KK Leiter LA Müller-Wieland D Cariou B Colhoun HM Henry RR . Alirocumab vs usual lipid-lowering care as add-on to statin therapy in individuals with type 2 diabetes and mixed dyslipidaemia: the ODYSSEY DM-DYSLIPIDEMIA randomized trial. Diabetes Obes Metab. (2018) 20:1479–89. doi: 10.1111/dom.13257, 29436756 PMC5969299

[32] Leiter LA Cariou B Müller-Wieland D Colhoun HM del Prato S Tinahones FJ . Efficacy and safety of alirocumab in insulin-treated individuals with type 1 or type 2 diabetes and high cardiovascular risk: the ODYSSEY DM-INSULIN randomized trial. Diabetes Obes Metab. (2017) 19:1781–92. doi: 10.1111/dom.13114, 28905478 PMC5698740

[33] Han Y Chen J Chopra VK Zhang S Su G Ma C . ODYSSEY EAST: alirocumab efficacy and safety vs. ezetimibe in high cardiovascular risk patients with hypercholesterolemia and on maximally tolerated statin in China, India, and Thailand. J Clin Lipidol. (2020) 14:98–108. doi: 10.1016/j.jacl.2019.10.015 31882376

[34] Kastelein JJ Ginsberg HN Langslet G Hovingh GK Ceska R Dufour R . ODYSSEY FH I and FH II: 78 week results with alirocumab treatment in 735 patients with heterozygous familial hypercholesterolaemia. Eur Heart J. (2015) 36:2996–3003. doi: 10.1093/eurheartj/ehv370, 26330422 PMC4644253

[35] Ginsberg HN Rader DJ Raal FJ Guyton JR Baccara-Dinet MT Lorenzato C . Efficacy and safety of alirocumab in patients with heterozygous familial hypercholesterolemia and LDL-C of 160 mg/dl or higher. Cardiovasc Drugs Ther. (2016) 30:473–83. doi: 10.1007/s10557-016-6685-y, 27618825 PMC5055560

[36] Koh KK Nam CW Chao TH Liu ME Wu CJ Kim DS . A randomized trial evaluating the efficacy and safety of alirocumab in South Korea and Taiwan (ODYSSEY KT). J Clin Lipidol. (2018) 12:162–172.e6. e6. doi: 10.1016/j.jacl.2017.09.007, 29153823

[37] Robinson JG Farnier M Krempf M Bergeron J Luc G Averna M . Efficacy and safety of alirocumab in reducing lipids and cardiovascular events. N Engl J Med. (2015) 372:1489–99. doi: 10.1056/NEJMoa1501031, 25773378

[38] Bays H Gaudet D Weiss R Ruiz JL Watts GF Gouni-Berthold I . Alirocumab as add-on to atorvastatin versus other lipid treatment strategies: ODYSSEY OPTIONS I randomized trial. J Clin Endocrinol Metab. (2015) 100:3140–8. doi: 10.1210/jc.2015-1520, 26030325 PMC4524987

[39] Farnier M Jones P Severance R Averna M Steinhagen-Thiessen E Colhoun HM . Efficacy and safety of adding alirocumab to rosuvastatin versus adding ezetimibe or doubling the rosuvastatin dose in high cardiovascular-risk patients: the ODYSSEY OPTIONS II randomized trial. Atherosclerosis. (2016) 244:138–46. doi: 10.1016/j.atherosclerosis.2015.11.010, 26638010

[40] Räber L Ueki Y Otsuka T Losdat S Häner JD Lonborg J . Effect of alirocumab added to high-intensity statin therapy on coronary atherosclerosis in patients with acute myocardial infarction: the PACMAN-AMI randomized clinical trial. JAMA. (2022) 327:1771–81. doi: 10.1001/jama.2022.5218, 35368058 PMC8978048

[41] Sabatine MS Giugliano RP Keech AC Honarpour N Wiviott SD Murphy SA . Evolocumab and clinical outcomes in patients with cardiovascular disease. N Engl J Med. (2017) 376:1713–22. doi: 10.1056/NEJMoa1615664, 28304224

[42] Nicholls SJ Puri R Anderson T Ballantyne CM Cho L Kastelein JJ . Effect of Evolocumab on progression of coronary disease in statin-treated patients: the GLAGOV randomized clinical trial. JAMA. (2016) 316:2373–84. doi: 10.1001/jama.2016.16951, 27846344

[43] Gaudet D Ruzza A Bridges I Maruff P Schembri A Hamer A . Cognitive function with evolocumab in pediatric heterozygous familial hypercholesterolemia. J Clin Lipidol. (2022) 16:676–84. doi: 10.1016/j.jacl.2022.07.005, 36210291

[44] Kastelein JJ Nissen SE Rader DJ Hovingh GK Wang MD Shen T . Safety and efficacy of LY3015014, a monoclonal antibody to proprotein convertase subtilisin/kexin type 9 (PCSK9): a randomized, placebo-controlled phase 2 study. Eur Heart J. (2016) 37:1360–9. doi: 10.1093/eurheartj/ehv707, 26757788 PMC4852062

[45] Ray KK Wright RS Kallend D Koenig W Leiter LA Raal FJ . Two phase 3 trials of inclisiran in patients with elevated LDL cholesterol. N Engl J Med. (2020) 382:1507–19. doi: 10.1056/NEJMoa1912387, 32187462

[46] Santanello NC Barber BL Applegate WB Elam J Curtis C Hunninghake DB . Effect of pharmacologic lipid lowering on health-related quality of life in older persons: results from the Cholesterol Reduction in Seniors Program (CRISP) pilot study. J Am Geriatr Soc. (1997) 45:8–14. doi: 10.1111/j.1532-5415.1997.tb00971.x, 8994481

[47] Carlsson CM Papcke-Benson K Carnes M McBride PE Stein JH. Health-related quality of life and long-term therapy with pravastatin and tocopherol (vitamin E) in older adults. Drugs Aging. (2002) 19:793–805. doi: 10.2165/00002512-200219100-00008, 12390056

[48] Li G Mayer CL Morelli D Millard SP Raskind WH Petrie EC . Effect of simvastatin on CSF Alzheimer disease biomarkers in cognitively normal adults. Neurology. (2017) 89:1251–5. doi: 10.1212/WNL.0000000000004392, 28821686 PMC5606918

[49] Criner GJ Connett JE Aaron SD Albert RK Bailey WC Casaburi R . Simvastatin for the prevention of exacerbations in moderate-to-severe COPD. N Engl J Med. (2014) 370:2201–10. doi: 10.1056/NEJMoa1403086, 24836125 PMC4375247

[50] Schanberg LE Sandborg C Barnhart HX Ardoin SP Yow E Evans GW . Use of atorvastatin in systemic lupus erythematosus in children and adolescents. Arthritis Rheum. (2012) 64:285–96. doi: 10.1002/art.30645, 22031171 PMC4074430

[51] Gupta A Thompson D Whitehouse A Collier T Dahlof B Poulter N . Adverse events associated with unblinded, but not with blinded, statin therapy in the Anglo-Scandinavian Cardiac Outcomes Trial-Lipid-Lowering Arm (ASCOT-LLA): a randomised double-blind placebo-controlled trial and its non-randomised non-blind extension phase. Lancet. (2017) 389:2473–81. doi: 10.1016/S0140-6736(17)31075-9, 28476288

[52] Marlatt KL Steinberger J Rudser KD Dengel DR Sadak KT Lee JL . The effect of atorvastatin on vascular function and structure in young adult survivors of childhood cancer: a randomized, placebo-controlled pilot clinical trial. J Adolesc Young Adult Oncol. (2019) 8:442–50. doi: 10.1089/jayao.2017.0075, 28853979 PMC6689188

[53] Fellström BC Jardine AG Schmieder RE Bannister K Beutler J Chae DW . Rosuvastatin and cardiovascular events in patients undergoing hemodialysis. N Engl J Med. (2009) 360:1395–407. doi: 10.1056/NEJMoa0810177 19332456

[54] Bosch J O’Donnell M Swaminathan B Lonn EM Sharma M Dagenais G . Effects of blood pressure and lipid lowering on cognition: results from the HOPE-3 study. Neurology. (2019) 92:e1435–46. doi: 10.1212/WNL.0000000000007174, 30814321 PMC6453765

[55] Ridker PM Danielson E Fonseca FA Genest J Gotto AM Jr Kastelein JJ . Rosuvastatin to prevent vascular events in men and women with elevated C-reactive protein. N Engl J Med. (2008) 359:2195–207. doi: 10.1056/NEJMoa0807646, 18997196

[56] Rossebø AB Pedersen TR Boman K Brudi P Chambers JB Egstrup K . Intensive lipid lowering with simvastatin and ezetimibe in aortic stenosis. N Engl J Med. (2008) 359:1343–56. doi: 10.1056/NEJMoa0804602, 18765433

[57] Tendolkar I Enajat M Zwiers MP van Wingen G de Leeuw FE van Kuilenburg J . One-year cholesterol lowering treatment reduces medial temporal lobe atrophy and memory decline in stroke-free elderly with atrial fibrillation: evidence from a parallel group randomized trial. Int J Geriatr Psychiatry. (2012) 27:49–58. doi: 10.1002/gps.2688, 21308791

[58] Kim BK Hong SJ Lee YJ Hong SJ Yun KH Hong BK . Long-term efficacy and safety of moderate-intensity statin with ezetimibe combination therapy versus high-intensity statin monotherapy in patients with atherosclerotic cardiovascular disease (RACING): a randomised, open-label, non-inferiority trial. Lancet. (2022) 400:380–90. doi: 10.1016/S0140-6736(22)00916-3, 35863366

[59] Engelberg H. Low serum cholesterol and suicide. Lancet. (1992) 339:727–9. doi: 10.1016/0140-6736(92)90609-7 1347593

[60] Orth M Bellosta S. Cholesterol: its regulation and role in central nervous system disorders. Cholesterol. (2012) 2012:292598. doi: 10.1155/2012/292598, 23119149 PMC3483652

[61] Donegá S Oba J Maranhão RC. Concentration of serum lipids and apolipoprotein B in newborns. Arq Bras Cardiol. (2006) 86:419–24. doi: 10.1590/s0066-782x2006000600003 16810415

[62] Faselis C Imprialos K Grassos H Pittaras A Kallistratos M Manolis A. Is very low LDL-C harmful? Curr Pharm Des. (2018) 24:3658–64. doi: 10.2174/1381612824666181008110643, 30295187

[63] Goldstein LB Toth PP Dearborn-Tomazos JL Giugliano RP Hirsh BJ Peña JM . Aggressive LDL-C lowering and the brain: impact on risk for dementia and hemorrhagic stroke: a scientific statement from the American Heart Association. Arterioscler Thromb Vasc Biol. (2023) 43:e404–42. doi: 10.1161/ATV.0000000000000164 37706297

[64] Bosco G Di Giacomo Barbagallo F Di Marco M Scilletta S Miano N Esposto S . Effect of inclisiran on lipid and mechanical vascular profiles in familial hypercholesterolemia subjects: results from a single lipid center real-world experience. Prog Cardiovasc Dis. (2025) 92:108–17. doi: 10.1016/j.pcad.2025.05.008, 40436259

[65] Wagstaff LR Mitton MW Arvik BM Doraiswamy PM. Statin-associated memory loss: analysis of 60 case reports and review of the literature. Pharmacotherapy. (2003) 23:871–80. doi: 10.1592/phco.23.7.871.32720, 12885101

[66] Pandor A Ara RM Tumur I Wilkinson AJ Paisley S Duenas A . Ezetimibe monotherapy for cholesterol lowering in 2,722 people: systematic review and meta-analysis of randomized controlled trials. J Intern Med. (2009) 265:568–80. doi: 10.1111/j.1365-2796.2008.02062.x, 19141093

[67] Ott BR Daiello LA Dahabreh IJ Springate BA Bixby K Murali M . Do statins impair cognition? A systematic review and meta-analysis of randomized controlled trials. J Gen Intern Med. (2015) 30:348–58. doi: 10.1007/s11606-014-3115-3, 25575908 PMC4351273

[68] Hirsh Raccah B Yanovsky A Treves N Rotshild V Renoux C Danenberg H . Proprotein convertase subtilisin/kexin type 9 (PCSK9) inhibitors and the risk for neurocognitive adverse events: a systematic review, meta-analysis and meta-regression. Int J Cardiol. (2021) 335:7–14. doi: 10.1016/j.ijcard.2021.04.025, 33892045

[69] Lipinski MJ Benedetto U Escarcega RO Biondi-Zoccai G Lhermusier T Baker NC . The impact of proprotein convertase subtilisin-kexin type 9 serine protease inhibitors on lipid levels and outcomes in patients with primary hypercholesterolaemia: a network meta-analysis. Eur Heart J. (2016) 37:536–45. doi: 10.1093/eurheartj/ehv563, 26578202

[70] Gencer B Mach F Guo J Im K Ruzza A Wang H . Cognition after lowering LDL-cholesterol with evolocumab. J Am Coll Cardiol. (2020) 75:2283–93. doi: 10.1016/j.jacc.2020.03.039, 32381158

[71] Santos RD Ruzza A Hovingh GK Wiegman A Mach F Kurtz CE . Evolocumab in pediatric heterozygous familial hypercholesterolemia. N Engl J Med. (2020) 383:1317–27. doi: 10.1056/NEJMoa2019910, 32865373

[72] Collins R Armitage J Parish S Sleight P Peto R Heart Protection Study Collaborative Group. Effects of cholesterol-lowering with simvastatin on stroke and other major vascular events in 20536 people with cerebrovascular disease or other high-risk conditions. Lancet. (2004) 363:757–67. doi: 10.1016/S0140-6736(04)15690-0, 15016485

[73] Sever PS Dahlöf B Poulter NR Wedel H Beevers G Caulfield M . Prevention of coronary and stroke events with atorvastatin in hypertensive patients who have average or lower-than-average cholesterol concentrations, in the Anglo-Scandinavian Cardiac Outcomes Trial-Lipid Lowering Arm (ASCOT-LLA): a multicentre randomised controlled trial. Lancet. (2003) 361:1149–58. doi: 10.1016/S0140-6736(03)12948-0 12686036

