# Supplementary material for: The effects of cholesterol-lowering drugs on neurocognitive function: systematic review and meta analysis
Source: Front Neurol. 2026 Feb 4;17:1696228. doi: 10.3389/fneur.2026.1696228 (PMC12913106; doi:10.3389/fneur.2026.1696228)
Supplement: Supplementary file 1 [file Supplementary_file_1.docx]

Appendix

Table 1 Pubmed Search strategy

| #1 | Hydroxymethylglutaryl-CoA Reductase Inhibitors[MeSH Terms] |
| --- | --- |
| #2 | HMG-CoA Reductase Inhibitor[Title/Abstract] |
| #3 | HMG-CoA Reductase Inhibitors[Title/Abstract] |
| #4 | Statin[Title/Abstract] |
| #5 | Statins[Title/Abstract] |
| #6 | Lovastatin[Title/Abstract] |
| #7 | Mevacor[Title/Abstract] |
| #8 | Altoprev[Title/Abstract] |
| #9 | Altocor[Title/Abstract] |
| #10 | Pravastatin[Title/Abstract] |
| #11 | Pravachol[Title/Abstract] |
| #12 | lipostat[Title/Abstract] |
| #13 | Simvastatin[Title/Abstract] |
| #14 | Zocor[Title/Abstract] |
| #15 | Fluvastatin[Title/Abstract] |
| #16 | Lescol[Title/Abstract] |
| #17 | Atorvastatin[Title/Abstract] |
| #18 | Lipitor[Title/Abstract] |
| #19 | Rosuvastatin[Title/Abstract] |
| #20 | Crestor[Title/Abstract] |
| #21 | Pitavastatin[Title/Abstract] |
| #22 | Livalo[Title/Abstract] |
| #23 | Ezetimibe[MeSH Terms] |
| #24 | Ezetimib[Title/Abstract] |
| #25 | Ezetrol[Title/Abstract] |
| #26 | Zetia[Title/Abstract] |
| #27 | SCH 58235[Title/Abstract] |
| #28 | Vytorin[Title/Abstract] |
| #29 | Inegy[Title/Abstract] |
| #30 | PCSK9 Inhibitors[MeSH Terms] |
| #31 | Proprotein Convertase Subtilisin Kexin Type 9 Inhibitors[Title/Abstract] |
| #32 | Proprotein Convertase Subtilisin Kexin Type 9 Inhibitor[Title/Abstract] |
| #33 | Alirocumab[Title/Abstract] |
| #34 | Praluent[Title/Abstract] |
| #35 | SAR236553[Title/Abstract] |
| #36 | REGN727[Title/Abstract] |
| #37 | Evolocumab[Title/Abstract] |
| #38 | Repatha[Title/Abstract] |
| #39 | AMG-145[Title/Abstract] |
| #40 | AMG145[Title/Abstract] |
| #41 | Inclisiran[Title/Abstract] |
| #42 | ALN-PCS[Title/Abstract] |
| #43 | ALN-PCSsc[Title/Abstract] |
| #44 | OR/ #1-43 |
| #45 | (randomized controlled trial[Publication Type] OR randomized[Title/Abstract] OR placebo[Title/Abstract]) |
| #46 | #44 AND #45 |
| #47 | [english]/lim |
| #48 | #46 AND #47 |

Table 2 Embase Search strategy

| #1 | 'hydroxymethylglutaryl coenzyme a reductase inhibitor'/exp |
| --- | --- |
| #2 | 'hmg-coa reductase inhibitor':ab,ti |
| #3 | 'hmg-coa reductase inhibitors':ab,ti |
| #4 | 'statin':ab,ti |
| #5 | 'statins':ab,ti |
| #6 | 'lovastatin':ab,ti |
| #7 | 'mevacor':ab,ti |
| #8 | 'altoprev':ab,ti |
| #9 | 'altocor':ab,ti |
| #10 | 'pravastatin':ab,ti |
| #11 | 'pravachol':ab,ti |
| #12 | 'lipostat':ab,ti |
| #13 | 'simvastatin':ab,ti |
| #14 | 'zocor':ab,ti |
| #15 | 'fluvastatin':ab,ti |
| #16 | 'lescol':ab,ti |
| #17 | 'atorvastatin':ab,ti |
| #18 | 'lipitor':ab,ti |
| #19 | 'rosuvastatin':ab,ti |
| #20 | 'crestor':ab,ti |
| #21 | 'pitavastatin':ab,ti |
| #22 | 'livalo':ab,ti |
| #23 | 'ezetimibe'/exp |
| #24 | 'ezetimib':ab,ti |
| #25 | 'ezetrol':ab,ti |
| #26 | 'zetia':ab,ti |
| #27 | 'sch 58235':ab,ti |
| #28 | 'vytorin':ab,ti |
| #29 | 'inegy':ab,ti |
| #30 | 'pcsk9 inhibitor'/exp |
| #31 | 'proprotein convertase subtilisin kexin type 9 inhibitors':ab,ti |
| #32 | 'proprotein convertase subtilisin kexin type 9 inhibitor':ab,ti |
| #33 | 'alirocumab':ab,ti |
| #34 | 'praluent':ab,ti |
| #35 | 'sar236553':ab,ti |
| #36 | 'regn727':ab,ti |
| #37 | 'evolocumab':ab,ti |
| #38 | 'repatha':ab,ti |
| #39 | 'amg-145':ab,ti |
| #40 | 'amg145':ab,ti |
| #41 | 'inclisiran':ab,ti |
| #42 | 'aln-pcs':ab,ti |
| #43 | 'aln-pcssc':ab,ti |
| #44 | OR/ #1-43 |
| #45 | 'randomized controlled trial'/exp |
| #46 | #44 AND #45 |
| #47 | [english]/lim |
| #48 | #46 AND #47 |

Table 3 Cochrane Library Search strategy

| #1 | MeSH descriptor: [Hydroxymethylglutaryl-CoA Reductase Inhibitors] explode all trees |
| --- | --- |
| #2 | (HMG-CoA Reductase Inhibitor):ti,ab,kw |
| #3 | (HMG-CoA Reductase Inhibitors):ti,ab,kw |
| #4 | (Statin):ti,ab,kw |
| #5 | (Statins):ti,ab,kw |
| #6 | (Lovastatin):ti,ab,kw |
| #7 | (Mevacor):ti,ab,kw |
| #8 | (Altoprev):ti,ab,kw |
| #9 | (Altocor):ti,ab,kw |
| #10 | (Pravastatin):ti,ab,kw |
| #11 | (Pravachol):ti,ab,kw |
| #12 | (lipostat):ti,ab,kw |
| #13 | (Simvastatin):ti,ab,kw |
| #14 | (Zocor):ti,ab,kw |
| #15 | (Fluvastatin):ti,ab,kw |
| #16 | (Lescol):ti,ab,kw |
| #17 | (Atorvastatin):ti,ab,kw |
| #18 | (Lipitor):ti,ab,kw |
| #19 | (Rosuvastatin):ti,ab,kw |
| #20 | (Crestor):ti,ab,kw |
| #21 | (Pitavastatin):ti,ab,kw |
| #22 | (Livalo):ti,ab,kw |
| #23 | MeSH descriptor: [Ezetimibe] explode all trees |
| #24 | (Ezetimib):ti,ab,kw |
| #25 | (Ezetrol):ti,ab,kw |
| #26 | (Zetia):ti,ab,kw |
| #27 | (SCH 58235):ti,ab,kw |
| #28 | (Vytorin):ti,ab,kw |
| #29 | (Inegy):ti,ab,kw |
| #30 | MeSH descriptor: [PCSK9 Inhibitors] explode all trees |
| #31 | (Proprotein Convertase Subtilisin Kexin Type 9 Inhibitors):ti,ab,kw |
| #32 | (Proprotein Convertase Subtilisin Kexin Type 9 Inhibitor):ti,ab,kw |
| #33 | (Alirocumab):ti,ab,kw |
| #34 | (Praluent):ti,ab,kw |
| #35 | (SAR236553):ti,ab,kw |
| #36 | (REGN727):ti,ab,kw |
| #37 | (Evolocumab):ti,ab,kw |
| #38 | (Repatha):ti,ab,kw |
| #39 | (AMG-145):ti,ab,kw |
| #40 | (AMG145):ti,ab,kw |
| #41 | (Inclisiran):ti,ab,kw |
| #42 | (ALN-PCS):ti,ab,kw |
| #43 | (ALN-PCSsc):ti,ab,kw |
| #44 | OR/ #1-43 |

Table 4 Clinicaltrials.gov Search strategy

| Intervention | Statin OR Ezetimibe OR (PCSK9 inhibitor) OR Alirocumab OR Evolocumab OR Inclisiran |
| --- | --- |
| Study Phase | (Phase 2) OR (Phase 3) OR (Phase 4) OR (Not applicable) |
| Study Type | Interventional |
| Study Results | With results |
